# Modeling the measles paradox reveals the importance of cellular immunity in regulating viral clearance

**DOI:** 10.1371/journal.ppat.1007493

**Published:** 2018-12-28

**Authors:** Sinead E. Morris, Andrew J. Yates, Rik L. de Swart, Rory D. de Vries, Michael J. Mina, Ashley N. Nelson, Wen-Hsuan W. Lin, Roger D. Kouyos, Diane E. Griffin, Bryan T. Grenfell

**Affiliations:** 1 Department of Ecology and Evolutionary Biology, Princeton University, Princeton, NJ, USA; 2 Department of Pathology and Cell Biology, Columbia University Medical Center, New York, NY, USA; 3 Department of Viroscience, Erasmus MC, Rotterdam, The Netherlands; 4 Department of Pathology, Brigham and Women’s Hospital, Harvard Medical School, Boston, MA, USA; 5 Department of Molecular Microbiology and Immunology, Johns Hopkins Bloomberg School of Public Health, Baltimore, MD, USA; 6 Division of Infectious Diseases and Hospital Epidemiology, University Hospital Zurich, Zurich, Switzerland; 7 Institute of Medical Virology, University of Zurich, Zurich, Switzerland; 8 Fogarty International Center, National Institutes of Health, Bethesda, MD, USA; Imperial College London, UNITED KINGDOM

## Abstract

Measles virus (MV) is a highly contagious member of the *Morbillivirus* genus that remains a major cause of childhood mortality worldwide. Although infection induces a strong MV-specific immune response that clears viral load and confers lifelong immunity, transient immunosuppression can also occur, leaving the host vulnerable to colonization from secondary pathogens. This apparent contradiction of viral clearance in the face of immunosuppression underlies what is often referred to as the ‘measles paradox’, and remains poorly understood. To explore the mechanistic basis underlying the measles paradox, and identify key factors driving viral clearance, we return to a previously published dataset of MV infection in rhesus macaques. These data include virological and immunological information that enable us to fit a mathematical model describing how the virus interacts with the host immune system. In particular, our model incorporates target cell depletion through infection of host immune cells—a hallmark of MV pathology that has been neglected from previous models. We find the model captures the data well, and that both target cell depletion and immune activation are required to explain the overall dynamics. Furthermore, by simulating conditions of increased target cell availability and suppressed cellular immunity, we show that the latter causes greater increases in viral load and delays to MV clearance. Overall, this signals a more dominant role for cellular immunity in resolving acute MV infection. Interestingly, we find contrasting dynamics dominated by target cell depletion when viral fitness is increased. This may have wider implications for animal morbilliviruses, such as canine distemper virus (CDV), that cause fatal target cell depletion in their natural hosts. To our knowledge this work represents the first fully calibrated within-host model of MV dynamics and, more broadly, provides a new platform from which to explore the complex mechanisms underlying *Morbillivirus* infection.

## Author summary

Measles is a highly contagious virus from the *Morbillivirus* genus that induces a strong adaptive immune response, despite also causing transient immunosuppression. How viral clearance is achieved amidst these seemingly contradictory dynamics (the so-called ‘measles paradox’) is poorly understood. To identify key factors driving viral clearance, we fit a mathematical model to data describing measles virus infection in rhesus macaques. In particular, our model incorporates target cell depletion through infection of immune cells—a hallmark of measles pathology neglected from previous models. The model accurately captures the data, and, by simulating effects of increased immune cell depletion and recovery, we find that cellular immunity is a stronger mediator of viral clearance than target cell depletion. Interestingly, we find contrasting dynamics dominated by target cell depletion when viral fitness is increased. This may have wider implications for closely related animal morbilliviruses that cause fatal target cell depletion in their natural hosts.

## Introduction

Measles virus (MV) is highly contagious and remains one of the leading causes of child mortality worldwide, despite the existence of a safe and effective vaccine [1]. In addition to its public health importance, MV is also a paradigm for understanding the dynamics of acute respiratory infections at broad epidemiological scales [2, 3]. However, at finer within-host scales, the kinetics of MV are less well understood. Unlike other common respiratory viruses such as influenza, MV does not initially infect epithelial cells of the respiratory tract [4]. Instead, MV is unusual in that it preferentially targets host immune cells, in particular those expressing the CD150 (SLAMF1) receptor [5, 6]. This atypical target population includes dendritic cells of the innate immune system, and B and T lymphocytes of the adaptive system [6, 7], and thus creates a dynamical feedback whereby the cells responsible for mounting an effective immune response are also under viral predation.

Clinical progression of disease initiates in the respiratory tract with infection of dendritic cells and alveolar macrophages [4]. These cells then migrate to lymph nodes and lymphoid tissues, where the virus replicates extensively in resident B and T lymphocytes, and eventually spreads systemically [8]. Therefore, although MV enters (and exits) the host via the respiratory tract, its subsequent pathogenesis is markedly different from that of classical respiratory viruses. During viral replication and spread, immune suppression coincides with the substantial depletion of host lymphocytes (typically from 7 days post infection (dpi) and recovering over a timescale of weeks) [8]. Most MV fatalities result from complications mediated by secondary infection during this period of immune vulnerability [9, 10]. However, in tandem there is also a rapid expansion of the adaptive immune system around 10–14 dpi, with activation of MV-specific effector T cells and increased antibody production [6, 11, 12]. These seemingly contrasting dynamics are often referred to as the ‘measles paradox’. Although the strong immune response ultimately leads to lifelong protection against reinfection, the interplay between immune cell depletion and activation, and its impact on viral clearance, are not well understood.

Conventional wisdom suggests that viral clearance is dominated by the adaptive immune response, in particular by MV-specific CD8^+^ cytotoxic T cells. Large expansions of these cells have been observed in humans during acute infection, and they have been shown to efficiently clear MV-infected cells *in vitro* [13–16]. In addition, patients with impaired cellular immunity are less able to control infection, experiencing delays to viral clearance and prolonged viral shedding [17]. These dynamics have also been tested *in vivo* through experimental infection of macaques, a model system for human infection [18–20]. For example, in Permar et al. (2003) macaques were depleted of CD8^+^ T cells prior to, and in the first four days following, MV infection. Compared to control individuals, macaques with depleted T cells experienced higher viral loads and delays to viral clearance from peripheral blood [21]. In contrast, macaques with impaired humoral immunity exhibit similar clinical pathology to control individuals [22]. Together, these findings suggest the CD8^+^ T cell response is an important factor limiting viral growth during peak viremia.

Despite this experimental work, however, questions still remain regarding the impact of target cell depletion on viral dynamics, particularly during early replication in lymphoid tissues. For example, MV is cytopathic and germinal centers of human patients show severe lymphoid exhaustion during the prodromal stages of infection; similar dynamics have also been observed in lymphoid tissues of macaques [8, 23–26]. Moreover, direct comparisons of viral kinetics in blood and lymphoid tissues have shown that target cells are generally infected at higher rates in the latter; for example up to 10% of peripheral B cells are infected during peak viremia, compared to over 30% in lymphoid tissues [6, 8]. The true extent of target cell depletion may therefore be underestimated in experiments analyzing only blood measurements. More generally, extreme target cell depletion has been observed during infections of closely related viruses within the same *Morbillivirus* genus. Ferrets infected with canine distemper virus (CDV) can experience infection of up to 80% of peripheral blood mononuclear cells (PBMC) during peak viremia and often succumb to disease before any immune response is detected [27]. Target cell limitation is therefore a major component of viral dynamics in similar eco-immunological systems.

Although both cellular immunity and target cell depletion may contribute to MV clearance, their relative importance has not been assessed systematically. Due to the complex dynamics of infection, with feedbacks between virus, target cells, and the host immune response, mathematical modeling provides a tractable approach to tease these drivers apart. Most notably, pioneering work modeling the dynamics of HIV (one of the best-studied lymphotropic viruses) has demonstrated the importance of both target cell depletion and host immunity in driving viral dynamics. For instance, simple ‘target cell limited’ models (i.e. models considering only the predation of virus on host target cells) can capture the early dynamics of infection [28–31], whereas incorporating CD8^+^ T cells can improve model predictions following the initial peak in viral load [30, 32–34]. However, key differences between HIV and MV are that the former infects CD4^+^ T cells and CD4-expressing macrophages and establishes lifelong infection, whereas the latter targets all CD150^+^ lymphocytes (including CD4^+^ and CD8^+^ T cells) and causes acute, self-limiting disease.

In general, fewer modeling studies have focused on acute infections due to the difficulty in generating high resolution data over the short timescales relevant for infection. Nonetheless, insights have been gained from particularly well-developed systems such as lymphocytic choriomeningitis virus (LCMV) and influenza. For example, models describing influenza A dynamics have revealed the importance of both the innate and adaptive immune responses in driving rapid viral turnover and eventual decline [35–37]. In comparison to LCMV and influenza, modeling acute MV infection poses the additional challenge that activated immune cells are also targets of viral predation. In particular, since MV-specific CD4^+^ and CD8^+^ effector T cells are susceptible to infection (although the latter at a potentially lower rate), their proliferation may counterintuitively facilitate viral growth by increasing the target cell population [13]. These potential activation costs have yet to be incorporated within a dynamic framework of MV infection [12, 38].

To our knowledge, two within-host models have previously been developed for MV infection, but neither accounted for the susceptibility of activated host immune cells. The first model, presented in Heffernan and Keeling (2008), assumed that target cells and MV-specific T cells were independent populations, and so the strength of the cellular immune response was unaffected by viral predation. Furthermore, although this framework captured some qualitative dynamics of infection, it was not calibrated against virological or immunological data and so could not be quantitatively verified. In contrast, the subsequent model of Lin et al. (2012) was calibrated against high resolution data and incorporated a wider array of adaptive immune mechanisms, including viral suppression by MV-specific antibodies and immune suppression by regulatory T cells. However, this model also ignored feedbacks between the virus and host immune cells, and thus did not fully capture the costs of immune activation during MV infection. In addition, the primary focus of the study was the long-term dynamics of MV within the host, rather than the clearance of acute viremia.

In this paper, we build on previous work by developing a new mathematical model of MV dynamics that incorporates predatory feedbacks between the virus and host immune cells. We calibrate the model using the virological and immunological data presented in Lin et al. (2012) and consequently find that a framework including both target cell depletion and cellular immune activation best captures the overall dynamics. By using the calibrated model to simulate the effects of increased target cell availability and suppressed cellular immunity, we find that the latter has a greater detrimental impact on the host’s ability to clear acute infection. This comparison provides quantitative evidence that cellular immunity is the more dominant factor driving viral clearance. Finally, by extending our analysis to incorporate higher viral growth rates, we demonstrate that the model can also reproduce extreme target cell depletion dynamics, such as those more in line with CDV infection in ferrets. In summary, this work presents the first calibrated model of MV dynamics to tease apart key drivers of viral clearance and, more generally, outlines a new quantitative framework to explore the complex dynamics underlying *Morbillivirus* infection.

## Results

### Experimental data

**Fig 1:**
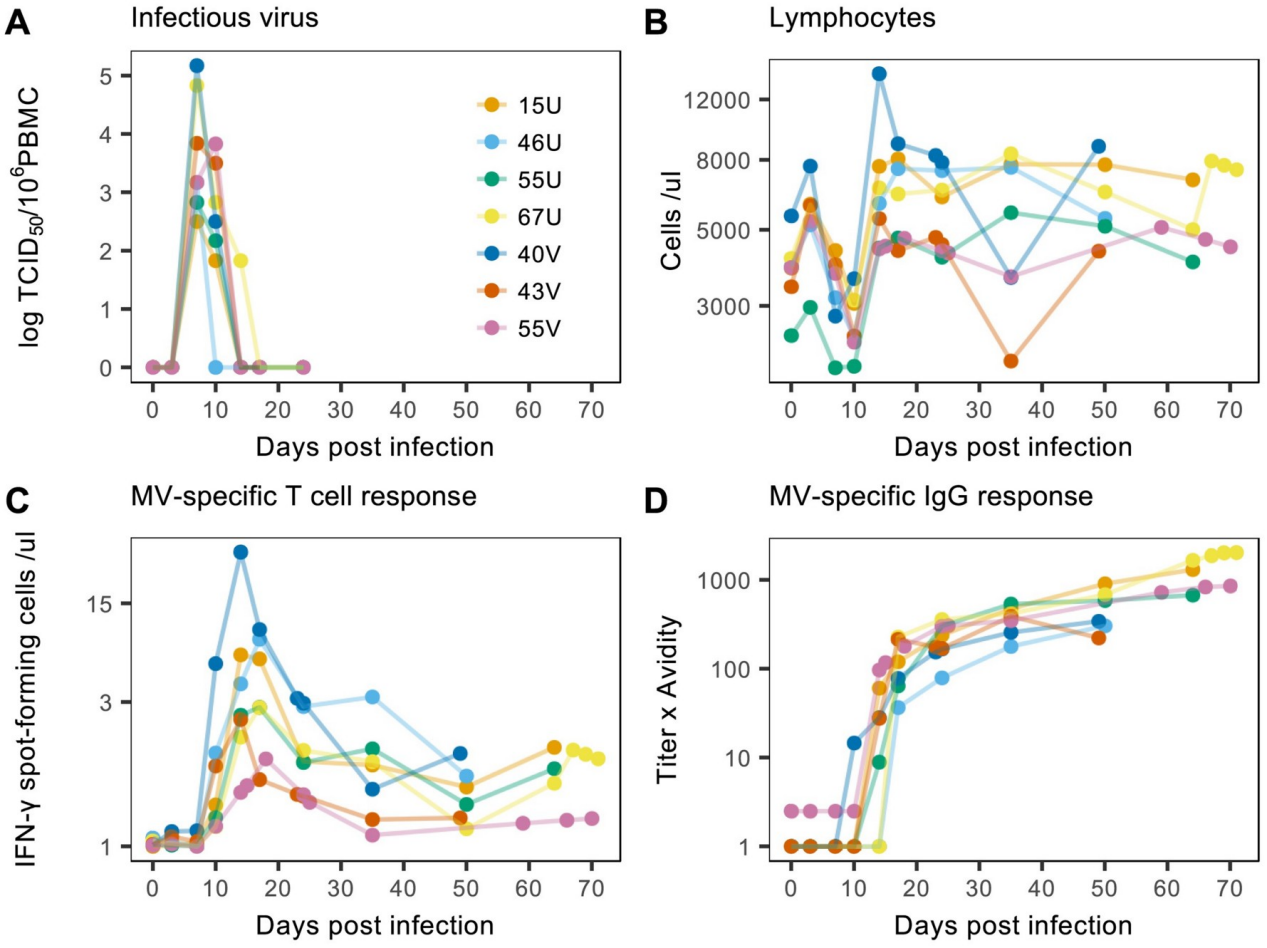
Immunological and virological data from Lin et al. (2012). Each color represents measurements from one of seven macaques (corresponding identification codes in panel A) infected with wild-type MV and monitored up to 71 days post infection. (A) Infectious viral load was determined by cocultivation of peripheral blood mononuclear cells with susceptible cells and then translated to tissue culture 50% infectious doses (TCID_50_) [40]. (B) The total number of lymphocytes per microliter of blood was measured using an automated cell counter. (C) MV-specific T cell activity was determined by the number of IFN-γ spot-forming cells per microliter of blood using enzyme-linked immunospot (ELISpot) assays. (D) MV-specific IgG titers were quantified using enzyme immunoassays (EIAs) and IgG avidity was measured by disruption of antibody binding [41]. Overall antibody response is expressed as IgG titer × avidity. Further details of the data and experimental methods are given in the S1 Appendix.

The data depict key virological and immunological components of MV infection (Fig 1) and have been described previously [12]. Briefly, seven juvenile rhesus macaques were infected with wild-type MV (Bilthoven strain) and monitored for up to 71 dpi. During the course of acute infection, regular blood samples were analyzed for infectious viral load, the number of circulating lymphocytes, and the activity of MV-specific T cells and antibodies. Despite substantial individual variation across the data, there are consistent and striking patterns. First, infectious viral load peaks between 7–10 dpi and coincides with a sharp decline in lymphocyte numbers, characteristic of MV-induced lymphophenia (Fig 1A and 1B). Following peak viral load, there is also a rapid increase in cellular and humoral immune activity, signifying the induction of a strong MV-specific adaptive immune response (Fig 1C and 1D). In particular, the T cell response peaks between 14–18 dpi and coincides with the recovery of the lymphocyte population, whereas the antibody response continues to increase past day 35. It is also important to note that since T cell activity was measured by *ex vivo* restimulation of PBMC, the data may underestimate the full potency of the original effector response [39]. Collectively, the distinct balance between viral growth, lymphocyte depletion, and subsequent immune cell stimulation typifies the measles paradox and demonstrates the utility of these data in exploring the role of target cell depletion and immune activation in driving viral clearance.

In addition to the classic characteristics of MV infection described above, we also note intriguing kinetics that are not well-described in previous literature. Firstly, at 3 dpi there is a striking and consistent increase in lymphocyte numbers across all individuals (Fig 1B). The biological mechanisms underlying this increase are not fully understood, but it may represent incoming lymphocytes from lymphoid tissues or early activation of peripheral lymphocytes. Secondly, after 35 dpi and the clearance of acute viremia, some individuals also experience a resurgence in lymphocyte and T cell numbers (e.g. 40V and 67U; Fig 1B and 1C). Again, the mechanisms for this recovery are not well-characterized, but it may signal the reactivation of MV-specific immune cells due to long-term persistence of infection [42–44]. We return to these points in the Discussion.

### Model selection and calibration

**Fig 2:**
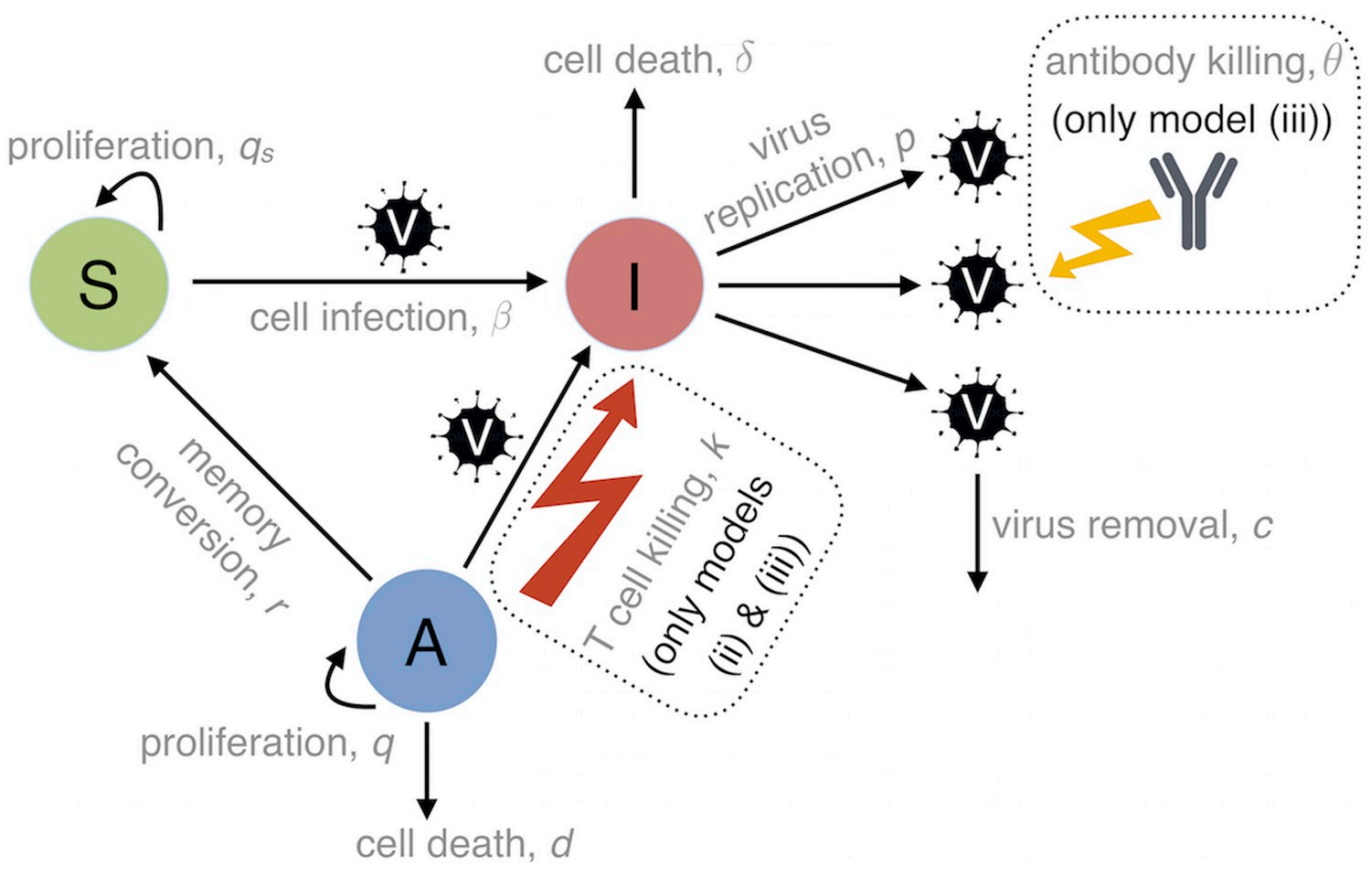
Schematic of the three main model structures: (i) target cell limited; (ii) target cells and T cells; and (iii) target cells, T cells, and antibodies. Virion shapes (*V*) represent infectious virus and circles represent distinct cell populations i.e. general susceptible lymphocytes (*S*), infected lymphocytes (*I*), and MV-specific activated T lymphocytes (*A*). Typical transitions of virion and cell dynamics are depicted by arrows and the constant biological parameters governing each transition are described by the corresponding text. The basic structure of model (i) is captured by the thin black arrows connecting all virion and cell populations. Model (ii) is obtained by the addition of T cell killing (red lightning bolt), and model (iii) is obtained by the addition of T cell killing and antibody killing (yellow lightning bolt). Additional functions governing the temporal evolution of T-cell activation and lymphocyte proliferation are discussed in the main text.

To examine the roles of target cells, T cells, and antibodies in driving within-host MV dynamics, three different models were fit to the immunological and virological data: (i) a target cell limited model, (ii) a model with target cells and activated T cells, and (iii) a model with target cells, activated T cells, and antibodies (see Materials and methods). Briefly, we assume host lymphocytes are the principal virus target cells, and divide these into subpopulations of ‘general’ susceptible cells (such as naive and memory B and T cells) and MV-specific T cells that become activated in response to viral load. Although epithelial cells in the respiratory tract and skin are infected following viremia, these are not considered primary drivers of virus dynamics and are thus excluded from the current model [6]. Initially, susceptible cells proliferate or become infected by virus. Infected cells then produce replicated virus which can infect other susceptible cells or is removed from the system through natural decay and non-specific immune processes. In model (iii), infectious virus is also cleared by antibodies. Infected cells are removed through natural decay and infection-induced apoptosis, and in models (ii) and (iii) are also killed by activated MV-specific T cells through direct lysis [11, 14, 15, 45, 46]. One crucial aspect of MV dynamics is that activated T cells may also be susceptible to viral predation. We therefore allow these cells to become infected, at which point they are assumed to lose all cytotoxic capabilities. For parsimony, we assume activated T cells are equally susceptible to infection as the general population, although in reality there may be nuances of heterogeneity across different lymphocyte subsets [8, 13]. Finally, activated T cells that escape infection eventually either die, or become memory T cells and join the general susceptible population. The model schematics are depicted in Fig 2 and the corresponding equations are presented in the Materials and methods.

**Fig 3:**
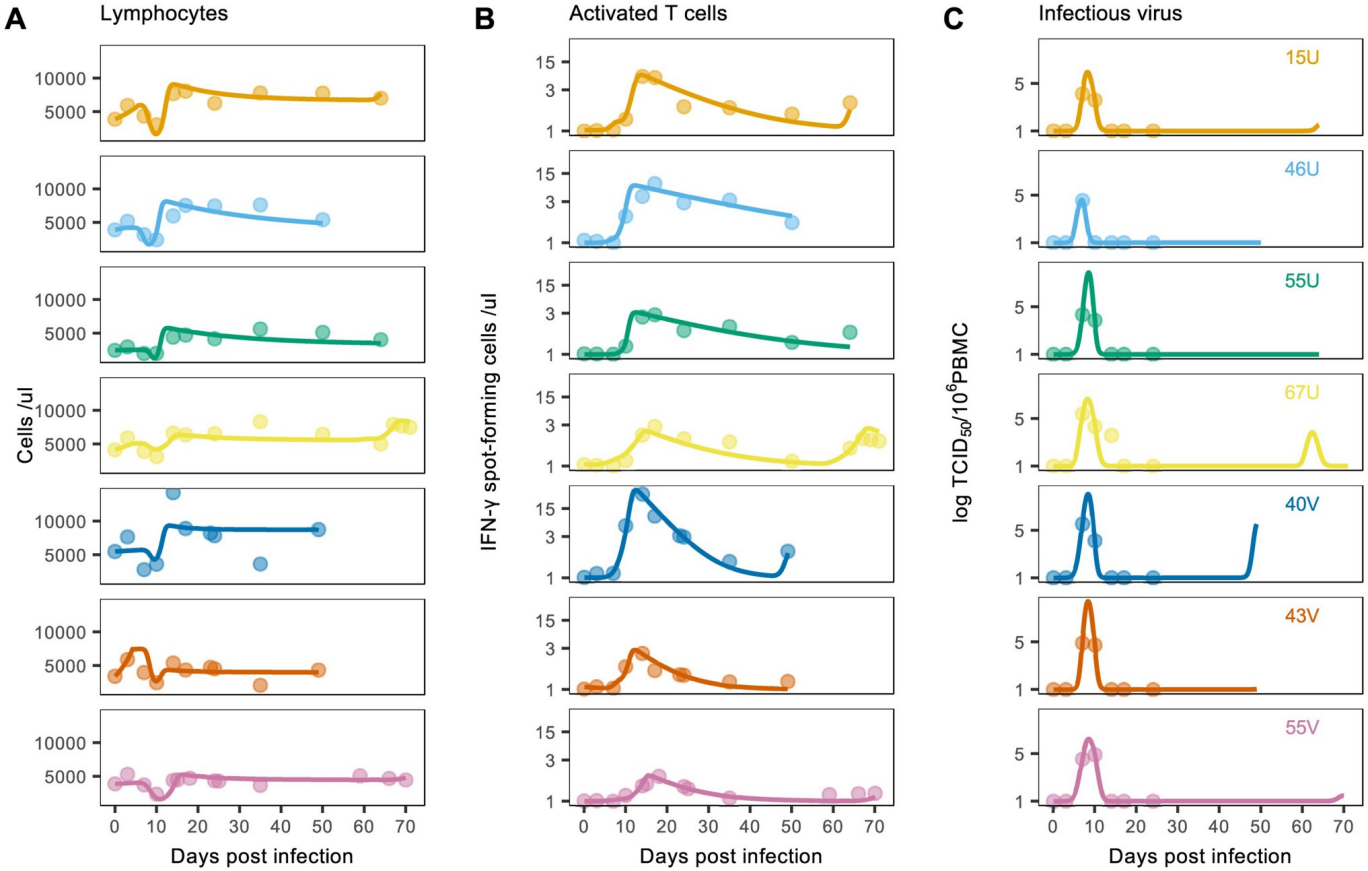
The target and T cell model calibrated with data from Lin et al. (2012). Points indicate data for (A) total lymphocytes, (B) MV-specific T cells, and (C) viral load; solid lines indicate the corresponding model predictions determined by maximum likelihood optimization. The activated T cell predictions are depicted before scaling for comparison with the MV-specific T cell data. Each row corresponds to an individual macaque (with identification codes inset in panel C), and panels B and C are shown on the log scale.

Each model was calibrated using the viral load, lymphocyte, and MV-specific T cell data (Fig 1A–1C), and statistical fits were compared using Akaike Information Criterion corrected for small sample sizes (AIC_c_; see Materials and methods). For the majority of individuals, AIC_c_ values indicated best support for model (ii) (Table 1), and the corresponding model predictions demonstrated close agreement with the data (Fig 3). Interestingly, this model also captured the late increases in lymphocyte and T cell numbers by predicting a corresponding resurgence in viral load (e.g. individual 67U; Fig 3). In contrast, the higher AIC_c_ values of model (iii) suggest the inclusion of antibodies did not provide a sufficient improvement in fit to warrant the increase in model complexity. Furthermore, although this model produced visually similar fits to the T cell model, it could not capture the late increase in cell numbers (S2 Fig). We therefore conclude that cellular immunity plays an important role in the acute dynamics of infection, whereas antibodies may be less relevant at these relatively short timescales. All subsequent analyses were thus conducted using the target cell and T cell model.

**Table 1:** Model comparisons using Akaike Information Criterion corrected for small sample sizes (AIC_c_).

Individual	(i) Target cells	(ii) Target cells and T cells	(iii) Target cells, T cells, and antibodies
15U	112.1	5.9	**0.0**
46U	64.2	**0.0**	2.9
55U	100.4	**0.0**	7.4
67U	21.5	**0.0**	16.1
40V	94.6	**0.0**	14.8
43V	102.3	**0.0**	1.9
55V	76.0	**0.0**	1.0

Models are listed in order of increasing complexity (from left to right). For each individual, the numerical values in each column indicate the difference in AIC_c_ between that model and the model with the lowest AIC_c_ (and hence best statistical support). Zero values (in bold) therefore indicate the best-supported model.

To test model parsimony with respect to target cell dynamics, we compared alternative functions governing the activity of MV-specific T cells and proliferation of the general susceptible population (see Materials and methods). For T cell activation, we chose a saturating function of viral load (Eq 5) that allows the activation and proliferation of T cells to dominate the immune response when viral load is high, and contraction and memory conversion to dominate once viral load has declined [47, 48]. Eq 5 has been previously employed for LCMV and has the advantage that T cell activation and contraction is described as a continuous dynamical process [47]. This function had greater statistical support than alternative expressions describing constant activation within a discrete time window, or activation that was proportional to viral load (S1 Table and S13 Fig). All subsequent analyses were therefore conducted using Eq 5. To capture the early activation of general lymphocytes, we explored a model in which proliferation was of temporary duration, *t*_*d*_ (where *t*_*d*_ was estimated during the fitting process) (Eq 6). Although simpler functions omitting proliferation, or allowing constant proliferation, had better statistical support (S2 Table and S14 Fig), the corresponding visual fits were relatively poor (see for example S3 Fig). In particular, the simpler models could not capture the initial increase in lymphocyte numbers observed at three dpi (S4 Fig). For the remainder of the paper we therefore use Eq 6, but note that this choice does not impact our general conclusions. For instance, when proliferation is omitted, we still find that the model with target cells and T cells outperforms the corresponding target cell only and target cell, T cell, and antibody models (S3 Table).

Parameter estimates for the best-fitting target cell and T cell model (Eqs 1–6) are presented in Table 2. As a measure of within-host viral fitness, the basic reproduction number (the average number of cells infected by one virus-infected cell in a fully susceptible population) was calculated from these estimates [49]. Note that here we distinguish between *R*_0_, the reproduction number in the absence of an initial immune response, and $R_{0}^{*}$, the corresponding expression when activated T cells are present. Despite variation amongst individuals, the parameters governing competition between viral growth and cellular immunity (e.g. *R*_0_, $R_{0}^{*}$, and the T cell proliferation and killing rates, *q* and *k*) were relatively well-conserved. To account for underestimation of the effector T cell response, a scaling factor, *ψ*, was included when fitting the model to the MV-specific T cell data (see Materials and methods). The resulting estimates were large and likely driven by the rapid recovery of total lymphocytes following lymphopenia. More specifically, since the predominant mechanism for lymphocyte expansion is through proliferation of activated T cells (*A*), capturing lymphocyte recovery required a greater expansion of these cells than was measured in the data. Finally, using fitted parameters from Table 2 we also estimated the half-life of an infected cell due to T cell killing, which is ln(2)/*kA* and changes over the course of infection. Across individuals, these half-lives ranged from 1.9–8.4 hours at peak viral load to 6.2–28 minutes at the height of the T cell response.

**Table 2:** Parameter estimates from the best-fitting model combining target cells and T cells.

Individual	Parameter													
	*R*_0_ ^†^	$R_{0}^{*}$ ^‡^	*S*_0_ ^§^	*A*_0_	*β*	*t*_*d*_	*k*	*p*	*q*	*q*_*s*_	*r*	*s*	*V*_0_	*ψ*
15U	6.9	5.3	3828	36.5	0.143	5.7	0.005	0.019	0.99	0.075	0.056	0.0104	1.1×10^-5^	992
46U	9.3	8.8	3905	1.1	0.165	2.6	0.024	0.022	1.11	0.028	0.016	0.0006	2.5×10^-5^	799
55U	8.2	7.8	2456	1.2	0.040	3.2	0.022	0.126	1.67	0.006	0.024	0.0993	1.0×10^-5^	1151
67U	15.9	3.7	4053	74.7	0.014	4.0	0.023	0.414	0.38	0.049	0.058	0.0007	1.0×10^-6^	959
40V	7.2	6.6	5487	2.6	0.013	7.0	0.017	0.152	1.11	0.006	0.159	0.0232	9.9×10^-5^	167
43V	6.9	2.2	3342	67.4	0.035	4.2	0.016	0.089	0.98	0.188	0.119	0.0997	3.0×10^-6^	585
55V	7.5	6.9	3876	3.8	0.056	7.0	0.011	0.052	0.59	0.007	0.089	0.0001	1.0×10^-4^	2206
**Median**														
	7.5	6.6	3876	3.8	0.040	4.2	0.017	0.089	0.99	0.028	0.058	0.0104	1.1×10^-5^	959
**Standard deviation**														
	3.2	2.4	906	32.9	0.062	1.8	0.007	0.137	0.41	0.066	0.052	0.0459	4.4×10^-5^	630

Median and standard deviations for each parameter were calculated using the estimates from all seven individuals. Parameter definitions are given in the Materials and methods. The values of *R*_0_, $R_{0}^{*}$, and *S*_0_ were calculated from the remaining eleven fitted parameters.

^†^*R*_0_ was calculated as *R*_0_ = *pβS*_0_/*cδ*, where *S*_0_ is the initial number of susceptible cells.

^‡^$R_{0}^{*}$ was calculated as $R_{0}^{*}=p\beta \left(S_{0}+A_{0}\right)/c\left(\delta +kA_{0}\right)$, where *A*_0_ is the initial number of activated T cells.

^§^*S*_0_ was calculated as *L*_0_ − *A*_0_, where *L*_0_ is the initial number of lymphocytes (obtained directly from the data).

To investigate the impact of parameter imprecision on model outcome, additional uncertainty and sensitivity analyses were conducted (see Materials and methods). Although the uncertainty analysis indicated substantial variation in model output for different parameter values (S7 Fig), subsequent sensitivity estimates suggested this was largely driven by a subset of key parameters, namely: the viral decay rate (*c*), the death rate of infected cells (*δ*), the viral replication rate (*p*), and, to a lesser extent, the T cell killing rate (*k*) (S8 Fig). By bootstrapping model residuals we also found parameters that were more conserved across individuals (e.g. *R*_0_, $R_{0}^{*}$, *q*) tended to have tighter confidence intervals, but a greater degree of correlation with other parameters (see for example S5 and S6 Figs). Together these results suggest that although many parameter combinations may provide similar qualitative fits to the data, the underlying balance between virus and immune cell competition is generally conserved.

### Drivers of viral clearance

**Fig 4:**
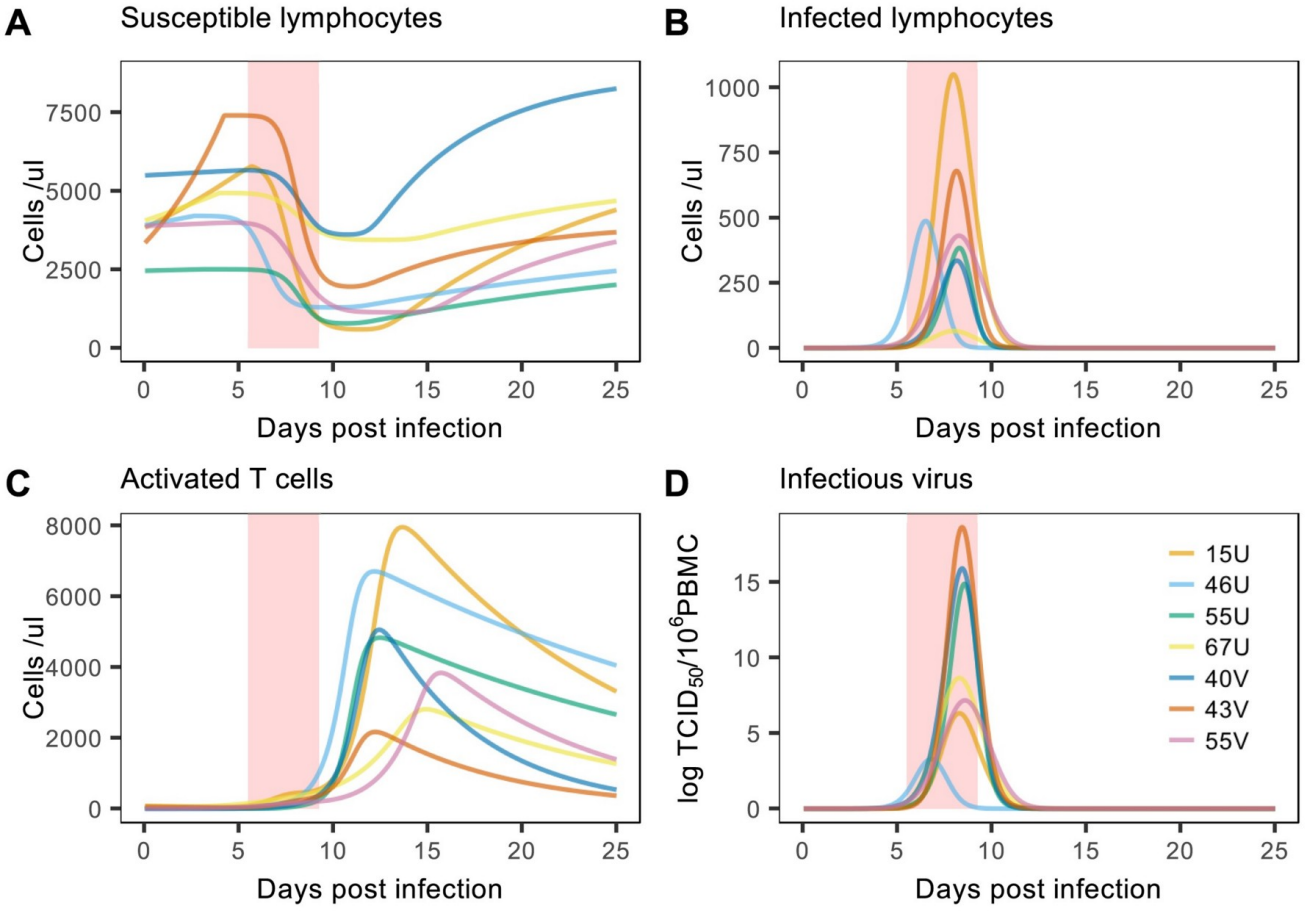
Predictions of the best-fitting target cell and T cell model. Solid lines indicate predictions for (A) susceptible lymphocytes, (B) infected lymphocytes, (C) activated T cells, and (D) viral load. Each color represents an individual macaque (with identification codes inset in panel D) and red shaded regions indicate the approximate window of peak viral load. Activated T cell numbers reflect absolute model predictions by incorporating the data scaling factor, *ψ*.

To explore drivers of viral clearance, we first qualitatively assessed the immunological and virological dynamics predicted by the best-fitting model. In all individuals, the sharp decline in susceptible target cells (up to 85%) coincided with the timing of peak viral load (shaded red regions, Fig 4A and 4D), and preceded the dramatic expansion of activated T cells (Fig 4C). One may therefore hypothesize that target cell depletion mediates viral dynamics during the initial turnover phase, before the cellular immune response is fully activated. Together with the previous AIC_c_ comparisons, these qualitative assessments suggest respective roles for both cellular immunity and target cell depletion in driving viral decline.

**Fig 5:**
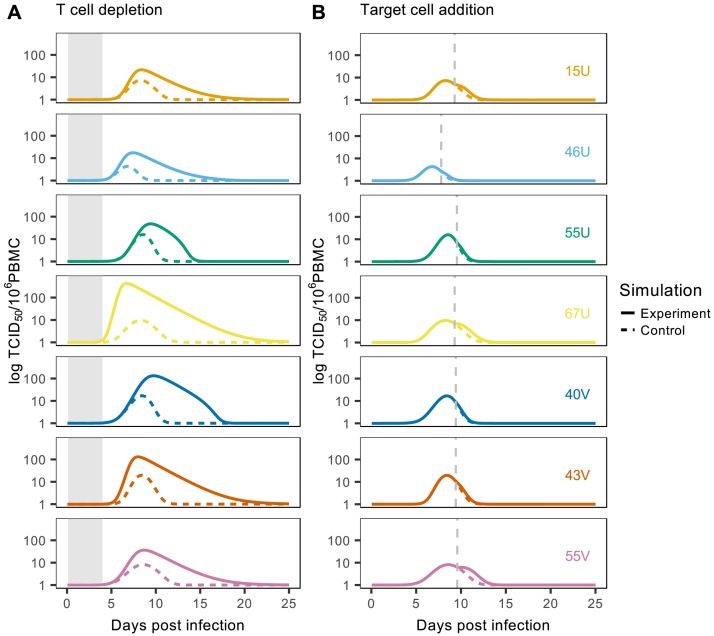
Investigating drivers of viral clearance. Solid lines indicate the effect of T cell depletion (A) or target cell addition (B) on viral load; dashed lines indicate the corresponding control simulations. Grey shaded regions in panel A represent the period of T cell depletion and the dashed grey vertical lines in panel B represent the timing of target cell addition. Each color represents an individual macaque (with identification codes inset in panel B).

To extend our qualitative assessments, we conducted two additional simulation experiments to quantitatively compare the impact of cellular immunity and target cell depletion on viral dynamics (see Materials and methods). First, to test the importance of cellular immunity, we recreated the T cell depletion experiment conducted by Permar et al. (2003). This involved reducing the initial population of activated T cells to low levels and then suppressing subsequent activation and proliferation until day four. Secondly, to test the importance of target cell depletion we simulated the addition of new susceptible cells to the lymphocyte pool one day after the occurrence of peak viral load. Results from both experiments were then compared to control simulations from the original model (i.e. Fig 4). In all cases we found that the depletion of activated T cells caused greater increases in peak viral load, and delays to viral clearance, than the addition of new target cells (Fig 5).

**Fig 6:**
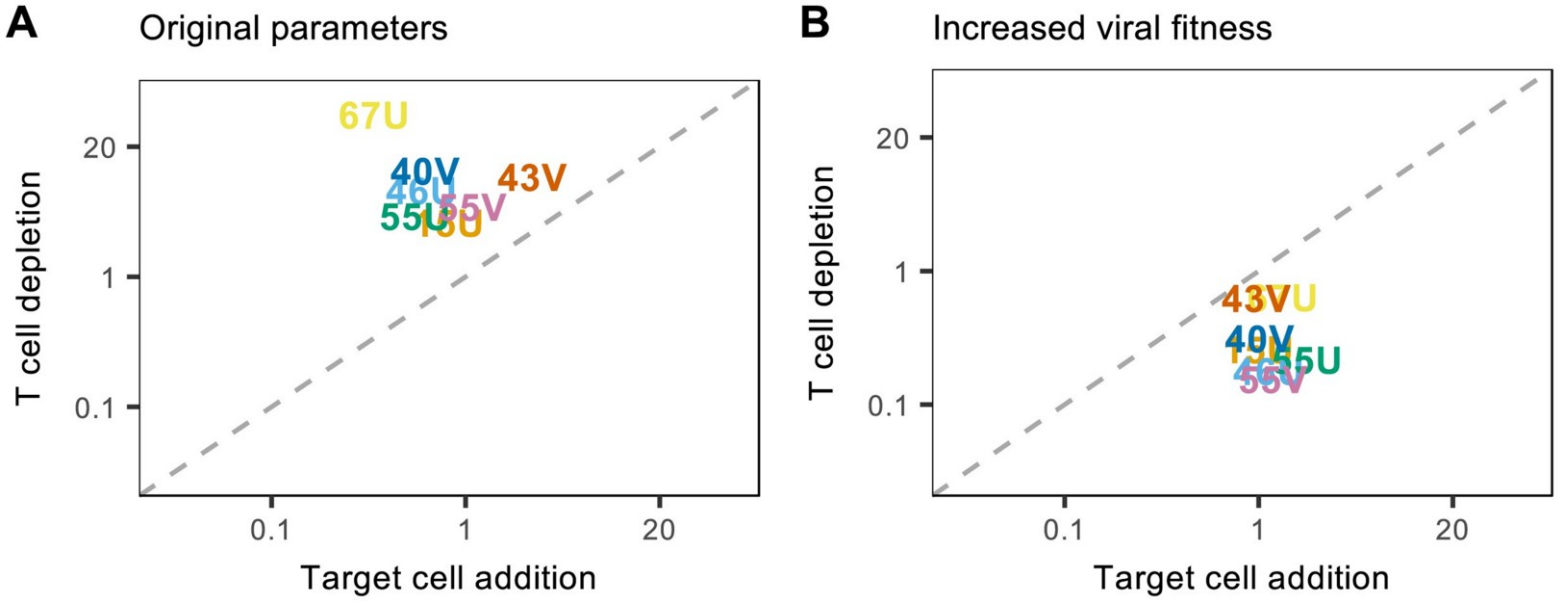
Comparing the dominant driver of viral clearance across individuals. For each macaque, the impacts of T cell depletion and target cell addition on viral load were calculated as the difference in the area under curve (AUC) between the experimental and control simulations, normalized by the AUC of the control simulation. Results for each individual are indicated by the corresponding identification code and the dashed line signifies the *y* = *x* boundary where experimental effects are equal. Simulations were conducted for (A) MV (by using best-fit parameters from the original target cell and T cell model); and (B) a virus with increased fitness (by doubling each individual’s viral replication rate, *p*). All axes are depicted on the log scale.

To systematically compare the impact of each simulation experiment across individuals, we measured the relative change in predicted viral load between each control and experimental simulation using Eq 7 (see Materials and methods and S1 Fig). For all macaques, T cell depletion resulted in a greater change in viral load than target cell addition, despite some individual variation in the magnitude of these changes (Fig 6A). These general findings were robust to changes in experimental conditions, including the initial number of activated T cells and duration of T cell depletion, and the magnitude and timing of target cell addition (S9 and S10 Figs). Our findings were also preserved when using the alternative lymphocyte proliferation functions that gained better statistical support (i.e. when we omitted proliferation, or allowed constant proliferation; see S15 Fig). Overall, these results suggest that cellular immunity is the more dominant factor driving clearance of acute MV infection.

Finally, we tested the versatility of the model by exploring its capacity to capture the extreme target cell depletion dynamics observed during other *Morbillivirus* infections, such as CDV. To do this, the above experiments were first repeated with an increased viral replication rate, *p* (see Materials and methods). This choice was guided by observations that viral load peaks earlier in macaques infected with CDV than MV, indicative of a higher viral growth rate [50]. Increasing the replication rate resulted in all individuals experiencing a greater impact of target cell addition than T cell depletion (Fig 6B), signaling a qualitative switch to dynamics driven by target cell limitation. Extending this analysis to other model parameters suggested that those relating to within-host viral fitness (i.e. the transmission rate, *β*, and the decay rate, *c*) and direct immune cell predation (the T cell killing rate, *k*) were also capable of producing this stark effect (S11 Fig). Although preliminary, these results suggest an important role for virus fitness phenotypes in determining ultimate drivers of *Morbillivirus* clearance.

## Discussion

In addition to its public health importance, MV is a paradigm for understanding the kinetics of acute immunizing infections at the epidemic scale [2, 3]. However, the dynamical underpinnings of MV immunity and immunosuppression are less well understood, in part due to a lack of integration between mathematical models and rich within-host data. We address this gap by developing a novel, data-rooted framework for modeling MV dynamics that incorporates predatory feedbacks between the virus and host immune cells. In general, we find that a model combining target cells and T cells best fits the data and is able to capture substantial variation in individual dynamics. By testing the relative impacts of T cell depletion and target cell addition on model predictions, we find that cellular immunity has a greater influence on viral clearance than target cell depletion. In addition to supporting the findings of previous experiments [21], this conclusion highlights the strength of MV-specific killer T cells in overcoming the direct effects of viral predation. More broadly, we find that the model can also reproduce dynamics dominated by target cell depletion (such as those observed for CDV) when viral growth rates are high or the strength of cellular immunity is low. Overall, our work provides a new quantitative platform from which to explore the complex dynamics of MV infection and test drivers of viral clearance across the *Morbillivirus* genus.

One caveat to the above conclusions is that our model is calibrated against data collected from the blood, and may not be representative of dynamics occurring elsewhere within the host. For example, higher proportions of infected cells are typically found in lymph nodes compared to circulating blood [6], suggesting that the former may experience greater amounts of target cell depletion and T cell activity than reflected in our data. Furthermore, resident lymphocytes in skin and respiratory submucosa show signs of infection for days following clearance from blood and lymphoid tissues, indicating potential modulation of host immunity in peripheral tissues [8, 51]. Together these observations imply differences in viral kinetics across multiple sites of infection. Further data comparing dynamics between the blood, lymphatic system, and peripheral tissues is needed to assess the impact of this spatial heterogeneity on our model predictions. However, experimental and ethical hurdles in obtaining such additional measurements make this a challenging prospect.

It is also important to note that it is unclear to what extent MV infection in lymphoid tissue is mediated by cell-cell contact or diffusing free virus [6, 52]. We explored alternative models in which infection occurs through direct cell-to-cell transmission, either with mass-action kinetics or with a modified stoichiometry to account for potential clustering effects [53]. Both models yielded substantially poorer fits (as determined by AIC_c_; see S1 Appendix, S4 and S5 Tables), suggesting that the flexibility afforded to the model by considering the dynamics of free virus is important. We speculate that this improvement, which derives from the virus density (*V*) roughly reflecting the integral of infected cell numbers over a timescale 1/*c* = 8 hours, represents (i) a significant role for infection by free virus diffusing in the lymphoid tissues and/or (ii) any time delay between infection of a cell and the onset of virus production. This latter point indicates that the infectious force at any instant is proportional to the recent history of infection events, rather than the instantaneous value of the infected cell density (*I*).

In addition to collecting spatially representative data, detecting the activity of MV-specific effector T cells is also experimentally challenging in macaques. Currently, *ex vivo* restimulation assays (such as those employed for the data in this study) provide the best measurements of T cell activity in nonhuman primates. However, capturing the kinetics of the total lymphocyte population required a large scaling of our T cell data, suggesting these underestimate the expansion of MV-specific T cells *in vivo*. One likely reason is that the assay measures T cells in peripheral blood, and may neglect the majority of cells fighting ongoing infections in lymphoid and peripheral tissues. Secondly, the assay requires *ex vivo* restimulation of cells that have been extensively stimulated *in vivo*, and may therefore reflect subsequent transitions to memory phenotype rather than true effector capacity [39]. On the other hand, the expansion of activated T cells may also be overestimated in our framework to compensate for other activation kinetics that are not explicitly modeled, such as the proliferation of other MV-specific and bystander lymphocytes [7, 8]. More accurately addressing these potential discrepancies between assay sensitivity and modeling assumptions is an important area for future work, and may be achieved by supplementing effector T cell data with measurements from lymphoid tissues, or additional data on B cell and non-specific lymphocyte responses.

To explore the role of adaptive immunity more fully, we also incorporated humoral immunity through the actions of MV-specific antibodies. However, in general we found better statistical support for a model without antibodies, and hypothesize that this is due to the relatively short timescale of acute viremia. Firstly, although the T cell and antibody data increase at similar times during the initial viral decline phase (10–20 dpi), the subsequent dynamics diverge as the former contract whereas the latter remain elevated. Consequently, the T cell population more closely follows the rise and fall of acute viral load, and thus provides a stronger signal for viral clearance. Secondly, we note that although the previous model of Lin et al. (2012) supported the inclusion of antibodies, it was calibrated using viral RNA data that persisted in the blood for 65 dpi and is thus more relevant over longer timescales of infection. Finally, experimental evidence suggests that T cells may be more influential than antibodies in clearing acute infection. For example, macaques with impaired humoral immunity exhibit similar clinical pathology to control individuals, whereas those with impaired cellular immunity experience higher viral loads and delays to clearance [21, 22]. Similar dynamics have also been observed in human patients [17, 54, 55]. Therefore, although antibodies are crucial for the establishment of long-term immunity, further work is required to resolve their role in suppressing acute viral dynamics [46].

In addition to the kinetics of acute viremia, we note that the model generates intriguing secondary peaks in viral load and lymphocyte numbers over longer timescales (around 50–60 dpi). By conducting subsequent sensitivity analysis, we found the time to viral resurgence is inversely associated with a subset of parameters, namely *A*_0_, *q*, and *r* (S1 Appendix and S17 Fig). The parameters *A*_0_ and *q* are associated with accelerated T cell activity, and *r* is associated with faster T cell contraction, suggesting that earlier viral control and resolution of immune activity facilitate more rapid virus rebound. Although our predictions of viral resurgence may be an artifact of the ordinary differential equation framework (whereby viral load can escape elimination despite falling below one infectious virion), we note that they mirror the resurgence in lymphocyte numbers observed in our data, and also echo qualitative viral dynamics observed in other macaque experiments [12, 42, 44]. In particular, Nelson et al. (2017) reported secondary peaks in viral RNA occurring at similar times to those predicted by our model (around 60 dpi). Such observations suggest persistent cycles of infection that are a hallmark of ecological predator-prey systems and may have implications for the recovery of the host immune system, and the development of lifelong MV-specific immunity. Although not the primary focus of this study, future work could explore these interactions further by integrating our model with longer term data on viral load and cell population dynamics. For instance, the development of MV-specific memory T cells could be modeled over the course of persistent infection by tracking activated T cells that transition to the susceptible compartment (as illustrated in the Materials and methods and S12 Fig).

One key challenge in modeling MV dynamics is that parameter values are poorly characterized compared to other systems such as HIV and LCMV. As a result, we fixed just three parameters prior to calibration, and subsequently tested model sensitivity to our fitted estimates. Firstly, we found substantial individual variation across the fitted estimates, and a number of parameter pairs that were highly correlated. Although this suggests many parameter combinations may provide similar qualitative fits, we did find certain values, such as those for *R*_0_, $R_{0}^{*}$, and the T cell proliferation rate, *q*, that were relatively well-conserved across individuals. In particular, *R*_0_ ranged from 6.9–15.9 (and $R_{0}^{*}$ from 2.2–8.8), similar to estimates for influenza (4.4–37.7) and HIV (3.8–11) [30, 35], and *q* reached 0.38–1.67 day^-1^, in line with previous CD8^+^ T cell expansion rates for HIV (0.51–1.22 day^-1^) [56]. Such conserved parameters provide benchmark estimates that should be validated in future work. In addition, sensitivity analysis suggested the majority of model uncertainty was driven by a subset of key parameters. Targeting these for future experimental estimation would allow more parameters to be fixed during model calibration and reduce problems of parameter correlations and identifiability.

In addition to *R*_0_ and *q*, we also found the half-life of an infected cell due to T cell killing was relatively consistent across individuals, reaching a minimum of 6.2–28 minutes at the height of the cellular immune response. Although very short, these are within ranges for acute LCMV infection (2–14 minutes) and reflect fast rates of T cell killing [57, 58]. This fast killing is linked to our assumption that the MV-specific T cell data predominantly reflect CD8^+^ T cells that mediate viral clearance through direct lysis of infected cells [11, 14, 15, 45, 46]. However, the data may also include CD4^+^ T cells that act through indirect, or ‘non-lytic’, mechanisms (for example secreting various cytokines that suppress viral replication) [12, 42]. While such additional mechanisms may impact the magnitude of our T cell killing rates during acute viral clearance, we expect the relative proportion of CD4^+^:CD8^+^ to remain approximately constant over time so that our qualitative conclusions are unchanged. Further disentangling indirect from direct killing mechanisms will be challenging with standard dynamic frameworks [59], particularly without data to distinguish between activated CD4^+^ and CD8^+^ cell subsets.

Beyond parameter estimation, our work also highlights the difficulty in accurately describing target cell dynamics. One such example is the increase in lymphocyte numbers during the initial days of infection. Although a model incorporating early lymphocyte proliferation produced better visual fits, it was statistically outperformed by a model ignoring this effect. Understanding the biological mechanisms underlying this dynamic will be crucial to developing a more parsimonious framework that is supported both qualitatively and quantitatively. Another example is that the current model does not account for heterogeneities within the lymphocyte population. In particular, we assume that all target cells are equally susceptible to infection, whereas evidence suggests that CD4^+^ T cells are more susceptible than CD8^+^ T cells *in vitro*, and memory lymphocytes are also preferentially targeted *in vivo* [8, 13]. Although the model could be adapted to distinguish between these different subsets, additional data regarding their relative abundance would first be required. Generating and integrating such data would be challenging given the substantial variation in lymphocyte phenotype distribution observed both within and between hosts [8].

Finally, to explore the ability of the model to capture dynamics dominated by target cell limitation rather than cellular immunity, we predominantly focussed on parameters mediating within-host viral fitness. This was motivated by observations that CDV causes extreme target cell depletion in natural host species (e.g. ferrets), and also reaches higher growth rates than MV during initial infection of macaques [27, 50, 60]. To simplify analysis, parameters were chosen to produce substantive yet biologically reasonable increases in viral fitness; for example, doubling *R*_0_ led to a range of 13.7–31.7 that was still within estimates for influenza. As the primary aim was to demonstrate the versatility of the model across a qualitative range of *Morbillivirus* dynamics, the analysis was necessarily approximate and did not incorporate a greater range of parameter modifications. However, in future work we intend to exploit this versatility to calibrate the model against comparative data for both MV and CDV. Such efforts will address important questions surrounding the mechanisms of viral clearance across different morbilliviruses.

### Conclusion

In conclusion, this paper presents the first within-host model of MV dynamics to incorporate predatory feedbacks between the virus and host immune system. By calibrating this model against virological and immunological data, we have provided insight into the roles of adaptive immunity and target cell depletion in mediating viral clearance. Although we note caveats associated with model parameterization and parsimony, these could be alleviated by future experimental advances and targeted data collection. In particular, efforts should focus on independent estimation of key parameters, and characterization of dynamics within different lymphocyte subsets. More broadly, comparing MV dynamics with CDV may provide further insights into mechanisms of viral clearance across the *Morbillivirus* genus.

## Materials and methods

### Model

The within-host target cell and T cell model is a system of ordinary differential equations given by

$$\begin{array}{c} \frac{dS}{dt}=-\beta SV+q_{s}\hat{\delta }\left(t\right)S+r\left(1-f\left(V\right)\right)A \end{array}$$ (1)

$$\begin{array}{c} \frac{dI}{dt}=\beta (S+A)V-\delta I-kIA \end{array}$$ (2)

$$\begin{array}{c} \frac{dA}{dt}=qf(V)A-\beta AV-(1-f(V))(d+r)A \end{array}$$ (3)

$$\begin{array}{c} \frac{dV}{dt}=pI-cV, \end{array}$$ (4)

where

$$\begin{array}{c} f\left(V\right)=\frac{V}{s+V} \end{array}$$ (5)

for some saturation constant, *s*, and

$$\begin{array}{c} \hat{\delta }\left(t\right)=\{\begin{array}{c} 1\;\;\;\text{if}\;\;\;t<t_{d},\\ 0\;\;\;\text{if}\;\;\;t\geq t_{d}. \end{array} \end{array}$$ (6)

As described above, host lymphocytes are initially divided into subpopulations of ‘general’ susceptible cells (*S*) and MV-specific activated T cells (*A*). The former includes naive and memory B and T cells, whereas the latter represents T cells that will become activated in response to viral infection. As the virus (*V*) enters the host, susceptible cells become infected (*I*) and produce replicated virus at per-capita rate *p*. Virus produced by these cells then infects more cells (at rate *βV*) or is removed from the system through natural decay and non-specific immune processes (*c*). During the first *t*_*d*_ days of infection, susceptible cells proliferate (*q*_*s*_) and, in response to viral load, MV-specific activated T cells proliferate (*q*) and kill infected cells through direct lysis (*k*). Infected cells are also removed from the system through natural and infection-induced decay (*δ*). Since activated T cells may also be susceptible to viral predation, we allow these cells to become infected, at which point they lose all cytotoxic capabilities. Finally, activated T cells that aren’t infected either die (*d*), or become memory T cells (*r*) and join the general susceptible population. The accumulation of these MV-specific memory T cells (*M*) can be predicted using the expression *dM*/*dt* = *r*(1 − *f*(*V*))*A*. Note that we assume the birth and death of susceptible cells over the short time span of acute viremia is small relative to infection-induced cell proliferation and death. We therefore omit these dynamics to preserve model parsimony.

The saturation function, *f*(*V*), was chosen to allow activation and proliferation of T cells to dominate the immunological response when viral load is high, and for contraction and memory conversion to dominate once viral load has declined [47, 48]. We also investigated alternative functions, including activation within a discrete timeframe and proliferation that was proportional to viral load (S1 Appendix and S13 Fig), but both resulted in poorer statistical fits to the data. We note that while infected cells produce both infectious and non-infectious virus particles, their relative roles in immune activation have yet to be determined. However, our general conclusions remain unchanged even if an (unknown) proportion of virus is non-infectious but still contributes to T cell activation. In this case, the saturation function is *f*(*αV*), where *α* is the (assumed constant) proportion of total virus that is immunostimulatory, and can be absorbed into the saturation parameter, *s*. Similarly, the proportion of virus that is infectious is contained within the transmission parameter, *β*.

The function governing lymphocyte proliferation, $\hat{\delta }\left(t\right)$, was chosen to capture the temporary increase in lymphocyte numbers arising from early activation. As the mechanisms underlying this dynamic are poorly understood, we also explored two simpler scenarios, one with constant proliferation (i.e. $\hat{\delta }\left(t\right)=1,\;\forall \;t$), and another with no proliferation (i.e. $\hat{\delta }\left(t\right)=0,\;\forall \;t$). Further details of these modifications can be found in the S1 Appendix and S14 Fig.

The model described by Eqs 1–6 incorporates the dynamics of target cell depletion and immune activity through the action of MV-specific killer T cells. However, we also investigated two alternative immunological scenarios. In the more complex scenario, we incorporated the humoral immune response by allowing MV-specific antibodies to provide additional viral clearance. This was achieved by modifying the viral load equation to

$$\frac{dV}{dt}=pI-cV-\theta YV,$$

where *Y*(*t*) represents the MV-specific IgG response (titer × avidity) at time *t*, and is input directly from the antibody data (Fig 1D). In the simpler scenario, we created a target cell limited model by removing all terms relating to direct immune-mediated viral clearance (i.e. the antibody clearance term, *θYV*, and the cytotoxic T cell term, *kIA*). In the absence of an adaptive immune response, the basic reproduction number for this model (the average number of cells infected by one virus-infected cell in a fully susceptible population) is given by *R*_0_ = *pβS*_0_/*cδ*, where *S*_0_ is the initial number of susceptible cells. However, when activated T cells are present at initial infection (i.e. *A*_0_ ≠ 0), $R_{0}^{*}=p\beta \left(S_{0}+A_{0}\right)/c\left(\delta +kA_{0}\right)$ [49]. Note that innate immunity was omitted from all models. In addition to preserving model parsimony, this choice was motivated by experimental evidence that MV inhibits innate immune functioning, in particular the production of IFN-α and IFN-β [61]. A list of all model parameters is given in Table 3 and the model schematics are illustrated in Fig 2.

**Table 3:** Parameters included in all model structures.

Parameter	Description	Units	Value
*A*_0_	Initial number of activated T cells	cells/μl	Fitted
*β*	Transmission rate	(log TCID_50_/10^6^ PBMC)^-1^ day^-1^	Fitted
*t*_*d*_	Duration of general lymphocyte activation	day	Fitted
*k*	Activated T cell killing rate	(cells/μl)^-1^ day^-1^	Fitted
*p*	Virus replication rate	(log TCID_50_/10^6^ PBMC)(cells/μl)^-1^ day^-1^	Fitted
*q*	Activated T cell proliferation rate	day^-1^	Fitted
*q*_*s*_	General lymphocyte proliferation rate	day^-1^	Fitted
*r*	Memory conversion rate	day^-1^	Fitted
*s*	Saturation parameter	log TCID_50_/10^6^ PBMC	Fitted
*V*_0_	Initial viral load	log TCID_50_/10^6^ PBMC	Fitted
*θ*	Rate of antibody-mediated clearance	(titer × avidity)^-1^ day^-1^	Fitted
*ψ*	Scaling factor for activated T cell data	-	Fitted
*c*	Viral load decay rate	day^-1^	3 [38]
*d*	Activated T cell death rate	day^-1^	1/40 [38]
*δ*	Infected cell death rate	day^-1^	1/2 [30, 31, 37]

### Statistical inference

The model was calibrated using the viral load, lymphocyte, and MV-specific T cell data (Fig 1A–1C). Specifically, the viral load data were used to fit the virus compartment (*V*), the total lymphocyte data to fit the sum of the model lymphocyte compartments (*S* + *I* + *A*), and the MV-specific T cell data to fit the activated T cell compartment (*A*). Since Lin et al. (2012) expressed the original T cell data in cells per million PBMC, these were first transformed to the scale of the lymphocyte data (cells per microliter) by using PBMC per microliter measurements from the original study. During the fitting process, the T cell data were also scaled by a constant, *ψ* ≥ 1, to account for underestimation of T cell activity during the *ex vivo* restimulation assay. For the model incorporating antibody-mediated viral clearance, we interpolated the MV-specific antibody data (Fig 1D) to approximate *Y*(*t*) at every time, *t*. Initial conditions for the total number of lymphocytes were obtained directly from the lymphocyte data, of which we assumed there were no infected cells (i.e. *I*(0) = *I*_0_ = 0). The initial viral load and number of activated T cells (*V*_0_ and *A*_0_, respectively) were estimated during the fitting process, and the initial number of susceptible cells (*S*_0_) was defined as the difference between the total number of lymphocytes and activated T cells. The differential equations of each model ((i) target cells only, (ii) target cells and T cells, and (iii) target cells, T cells, and antibodies), were solved numerically in R using the deSolve package [62]. Following previous studies, the subsequent viral load, activated T cell, and total lymphocyte predictions were fit to the corresponding data by maximum likelihood optimization in R, assuming normally-distributed residuals between the log-transformed data and model predictions [36, 37, 63]. Results in the main text are reported from models fit independently to each individual. However, fitting the models to pooled data across all individuals gave qualitatively similar results (e.g. S16 Fig). Further details of the fitting procedure can be found in the S1 Appendix.

The statistical support for each model was compared using Akaike Information Criterion corrected for small sample sizes (AIC_c_) [64, 65]. This metric assesses goodness of fit whilst also penalizing models with more parameters. The AIC_c_ value for each model is given by

$$\begin{array}{c} {\text{AIC}}_{c}=2\hat{k}-2\ln\hat{L}+\frac{2\hat{k}\left(\hat{k}+1\right)}{n-\hat{k}-1}, \end{array}$$

where $\hat{L}$ is the maximum likelihood estimate, $\hat{k}$ is the number of estimated parameters, and *n* is the number of data points available for fitting. Lower AIC_c_ values indicate a more parsimonious fit and were thus used to identify the best-fitting model.

Approximate credible intervals on parameter estimates were obtained by bootstrapping model residuals with 500 replicates. These replicates were also used to estimate pairwise parameter correlations. Additional uncertainty and sensitivity analyses were undertaken using Latin Hypercube sampling (LHS) and partial rank correlation (PRC) methods [66]. In combination, these techniques assess how imprecision in input parameters impacts model predictions, and can identify parameters with the greatest influence on model outcome. Such methods have been described previously with respect to HIV transmission models [66], and further details of the implementation in this study are given in the S1 Appendix.

### Drivers of viral clearance

To compare the impact of target cell depletion and cellular immune activity on viral clearance we conducted two additional simulation experiments with the best-fitting model. First, to test the importance of cellular immunity we recreated the T cell depletion experiment conducted by Permar et al. (2003). Specifically, we reduced the initial population of activated T cells to low levels (*A*_0_ = 1×10^-1^ cells/μl) and then suppressed subsequent activation and proliferation until day four. Results were then compared to control simulations with no restrictions on T cell activity. We hypothesized that a delay to viral clearance, as observed in the original experiments [21], would signal an important role for cellular immunity in mediating viral decline. Secondly, to test the importance of target cell depletion we performed additional simulations in which new susceptible cells (*S*_*new*_ = 2000) were added to the lymphocyte pool one day after the occurrence of peak viral load. We hypothesized that if target cell availability limits viral growth and contributes to eventual decline, adding new cells should release this restriction and allow viral load to increase again.

To compare the impact of each simulation experiment across individuals, we measured the relative change in predicted viral load, Δ_*V*_. This was defined as the difference in the area under the curve (AUC) between the experimental (*V*_*E*_) and control (*V*_*C*_) simulations, normalized by the AUC of the control simulation i.e.

$$\begin{array}{c} \Delta_{V}=\frac{\int_{0}^{t_{T}}V_{E}\;dt-\int_{0}^{t_{T}}V_{C}\;dt}{\int_{0}^{t_{T}}V_{C}\;dt}, \end{array}$$ (7)

where *t*_*T*_ is the total simulation time (for an illustration see S1 Fig). To test the sensitivity of our results to experimental conditions, we repeated the above analysis whilst varying key parameters in each simulation. For the T cell depletion experiment we changed the initial number of activated T cells and the duration of T cell suppression, and for the target cell addition experiment we varied the number of new target cells added and the timing of this addition.

Finally, we tested the versatility of the model by exploring its capacity to capture the extreme cell depletion dynamics observed in other *Morbillivirus* systems, such as CDV. More specifically, we repeated the above analysis whilst changing certain parameter conditions to favor viral fitness over cellular immunity. This choice was guided by observations that viral load peaks earlier in macaques infected with CDV than MV, indicative of a higher viral growth rate [50]. For each new condition, one parameter from the original best-fitting model was modified, and the control and experimental simulations were performed as described above. Recalculating Δ_*V*_ then provided a new comparison of the impact of target cell depletion and cellular immunity on viral clearance. To simplify the analysis we first chose parameter modifications that would double *R*_0_, and therefore increase the within-host viral fitness by a biologically substantive degree. This included scenarios which doubled the replication rate, *p*, doubled the transmission rate, *β*, or halved the viral decay rate, *c*. For comparative purposes, the investigation was then extended to other parameters mediating the strength of cellular immunity. This included halving the T cell killing rate, *k*, and proliferation rate, *q*. Parameters that led to the greatest influence of target cell depletion on viral dynamics were identified from the final set of comparisons.

## Supporting information

S1 Fig Quantification of the relative change in viral load using individual 55U as an example. In each panel, upper green lines indicate the treatment effect ((A) T cell depletion or (B) target cell addition) and lower green lines indicate the corresponding control simulation. The effect of each treatment is calculated as the difference in the area under the curve (AUC) between the treatment and control simulations (green shaded regions), normalized by the AUC of the control simulation (white regions). The grey rectangular region represents the period of T cell depletion and the dashed grey vertical line represents the timing of target cell addition.
(PDF)

S2 Fig The target cell, T cell, and antibody model calibrated with data from Lin et al. (2012). Points indicate data for (A) total lymphocytes, (B) MV-specific T cells, and (C) viral load; solid lines indicate the corresponding model predictions determined by maximum likelihood optimization. The activated T cell predictions are depicted before scaling for comparison with the MV-specific T cell data. Each row corresponds to an individual macaque (with identification codes inset in panel C), and panels B and C are shown on the log scale.
(PDF)

S3 Fig The target cell and T cell model without lymphocyte proliferation, calibrated with data from Lin et al. (2012). Points indicate data for (A) total lymphocytes, (B) activated T cells, and (C) viral load; solid lines indicate the corresponding model predictions determined by maximum likelihood optimization. The activated T cell predictions are depicted before scaling for comparison with the MV-specific T cell data. Each row corresponds to an individual macaque (with identification codes inset in panel C), and panels B and C are shown on the log scale.
(PDF)

S4 Fig Comparison of alternative general lymphocyte proliferation functions. Solid lines indicate lymphocyte dynamics predicted by the target cell and T cell model without lymphocyte proliferation (blue) and with early lymphocyte proliferation (orange); points indicate lymphocyte data from Lin et al. (2012). Each panel corresponds to an individual macaque (indicated by the panel label).
(PDF)

S5 FigRepresentative parameter confidence intervals from individual 55V.Histograms show fitted parameter estimates obtained from 500 bootstrap samples. *R*_0_ was calculated from the fitted parameters as *pβS*_0_/*cδ*; similarly $R_{0}^{*}$ was calculated as *pβ*(*S*_0_ + *A*_0_)/*c*(*δ* + *kA*_0_). Shaded regions encompass the 90th percentiles.(PDF)

S6 Fig Representative pairwise parameter correlations from individual 55V. Fitted parameter estimates were obtained from 500 bootstrap samples. Colors and numerical values indicate the magnitude of correlation; correlations above a standard significance threshold (i.e. *p* > 0.05) are depicted in white.
(PDF)

S7 Fig Uncertainty analysis for the target cell and T cell model. Each point represents the output (summarized here as total viral load) obtained from 1 of 100 different parameter sets generated by Latin Hypercube sampling. The corresponding distributions and box plots for each individual are outlined in black.
(PDF)

S8 Fig Partial rank correlation coefficient analysis to assess sensitivity of the target cell and T cell model. Each bar represents a different parameter, and the absolute height represents the magnitude of model sensitivity to that parameter. Positive values indicate that an increase in parameter value causes a positive change in the measured model output (i.e. an increase in total viral load), whereas negative values indicate a negative change. Note that the scaling factor, *ψ*, was omitted from this analysis as it does not appear in the model equations or directly impact the resulting predictions. Each panel corresponds to an individual macaque (identified by the panel label). Significance thresholds are defined as follows: *p < 0.05, **p < 0.01, ***p < 0.001.
(PDF)

S9 Fig Sensitivity of the T cell depletion simulation to experimental conditions. The relative change in viral load (or ‘relative effect’) was recalculated whilst: (A) the initial number of activated T cells (*A*_0_) was varied between 0.01, 0.1 (original condition), and 1 cells/μl; and (B) the duration of T cell suppression was varied between 3, 4 (original condition), and 5 days. On each panel, the effect of the original target cell addition experiment (‘Target cells’) is shown for comparison. For each macaque, the relative change in viral load was calculated as the difference in the area under the curve (AUC) between the experimental and control simulations, normalized by the AUC of the control simulation. Results for each individual are indicated by the corresponding identification code. In all but one case (individual 55U when *A*_0_ = 1 cells/μl), the effect of T cell depletion for each individual remains greater than that of target cell addition.
(PDF)

S10 Fig Sensitivity of the target cell addition simulation to experimental conditions. The relative change in viral load (or ‘relative effect’) was recalculated whilst: (A) the number of added target cells was varied between 1000, 2000 (original condition), and 3000 cells/μl; and (B) the timing of target cell addition was varied between 0.5, 1 (original condition), and 1.5 days following peak viral load. On each panel, the effect of the original T cell depletion experiment (‘T cells’) is shown for comparison. For each macaque, the relative change in viral load was calculated as the difference in the area under the curve (AUC) between the experimental and control simulations, normalized by the AUC of the control simulation. Results for each individual are indicated by the corresponding identification code. In all cases the effect of target cell addition for each individual remains less than that of T cell depletion.
(PDF)

S11 Fig Relative effect of T cell depletion and target cell addition for different parameter modifications. For each macaque, the relative effect of each experiment is calculated as the difference in the area under the viral load curve (AUC) between the experimental and control simulations, normalized by the AUC of the control simulation. Results for each individual are indicated by the corresponding identification code. Parameters from the original target cell and T cell model are given in panel A. All other panels represent the impact of changing one parameter (indicated by the panel title). Panels (B)–(F) represent changes that cause *R*_0_ = *pβS*_0_/*cδ*, the within-host viral fitness, to double. Panels (G)–(I) represent changes that reduce the strength of the T cell response. Panels (J)–(L) represent other parameter changes that are not directly related to viral fitness or T cell predation.
(PDF)

S12 Fig Cumulative development of MV-specific memory T cells. For each macaque, the development of MV-specific memory T cells can be predicted by tracking the activated T cells that transition to the susceptible cell compartment. Solid lines indicate the predictions of the best-fitting target cell and T cell model, and each color represents an individual macaque (with identification codes in the inset legend).
(TIF)

S13 Fig Functions governing T cell activation. Three different functions are used to model the activation of MV-specific T cells, *f*(*V*): (A) activation that is proportional to viral load; (B) activation that saturates at high viral loads; and (C) constant activation within a discrete timeframe. Solid lines indicate *f*(*V*) for each model, and each color represents an individual macaque (with identification codes in panel C). Mathematical formulae for *f*(*V*) are given in the Materials and methods and S1 Appendix.
(TIF)

S14 FigFunctions governing lymphocyte proliferation.Three different functions are used to model the proliferation of susceptible lymphocytes, $\hat{\delta }\left(t\right)$: (A) no proliferation; (B) constant proliferation; and (C) constant proliferation within a temporary timeframe. Solid lines indicate $\hat{\delta }\left(t\right)$ for each model, and each color represents an individual macaque (with identification codes in panel C). Mathematical formulae for $\hat{\delta }\left(t\right)$ are given in the Materials and methods and S1 Appendix.(TIF)

S15 FigComparing drivers of viral clearance with alternative lymphocyte proliferation functions.Three different functions are used to model the proliferation of susceptible lymphocytes, $\hat{\delta }\left(t\right)$: (A) no proliferation; (B) constant proliferation; and (C) constant proliferation within a temporary timeframe. For each macaque, the impacts of T cell depletion and target cell addition on viral load were calculated as the difference in area under curve (AUC) between the experimental and control simulations, normalized by the AUC of the control simulation. Results for each individual are indicated by the corresponding identification code and the dashed line signifies the *y* = *x* boundary where experimental effects are equal. Mathematical formulae for all proliferation functions are given in the Materials and methods and S1 Appendix.(PDF)

S16 Fig Comparing the drivers of viral clearance between the pooled and individual fits. For each individual (or pooled) fit, the impacts of T cell depletion and target cell addition on viral load were calculated as the difference in area under curve (AUC) between the experimental and control simulations, normalized by the AUC of the control simulation. Results for each individual are indicated by the corresponding identification code and the dashed line signifies the *y* = *x* boundary where experimental effects are equal. Results for the pooled data are indicated by the grey ‘Pooled’ label. Simulations were conducted for (A) MV (by using best-fit parameters from the original target cell and T cell model); and (B) a virus with increased fitness (by doubling the viral replication rate, *p*, of each individual (or pooled) fit).
(PDF)

S17 Fig Partial rank correlation coefficient analysis to assess sensitivity of the predicted time to rebound in the best fitting model. Each bar represents a different parameter, and the absolute height represents the magnitude of model sensitivity to that parameter. Positive values indicate that an increase in parameter value causes a positive change in the measured model output (i.e. the time to virus rebound), whereas negative values indicate a negative change. Note that the scaling factor, *ψ*, was omitted from this analysis as it does not appear in the model equations or directly impact the resulting predictions. Each panel corresponds to an individual macaque (identified by the panel label). Significance thresholds are defined as follows: *p < 0.05, **p < 0.01, ***p < 0.001.
(PDF)

S1 Appendix Further details of experimental data, model formulations, and fitting procedures. (PDF)

S1 Table Comparison of alternative T cell activation functions using AIC_c_. Each row represents a different individual and columns represent different model structures (in order of increasing complexity from left to right). For each individual, numerical values indicate the difference in AIC_c_ between each model and the model with the lowest AIC_c_ (and hence best statistical support). Zero values (in bold) therefore indicate the best-supported model.
(PDF)

S2 Table Comparison of alternative general lymphocyte proliferation functions using AIC_c_. Each row represents a different individual and columns represent different model structures (in order of increasing complexity from left to right). For each individual, numerical values indicate the difference in AIC_c_ between each model and the model with the lowest AIC_c_ (and hence best statistical support). Zero values (in bold) therefore indicate the best-supported model.
(PDF)

S3 TableModel comparisons using AIC_c_ when general lymphocyte proliferation is omitted (i.e. $\hat{\delta }\left(t\right)=0\;\forall \;t$).Each row represents a different individual and columns represent different model structures (in order of increasing complexity from left to right). For each individual, numerical values indicate the difference in AIC_c_ between each model and the model with the lowest AIC_c_ (and hence best statistical support). Zero values (in bold) therefore indicate the best-supported model.(PDF)

S4 Table Comparison of alternative transmission kinetics using AIC_c_. Each row represents a different individual and columns represent different model structures (in order of increasing complexity from left to right). For each individual, numerical values indicate the difference in AIC_c_ between each model and the model with the lowest AIC_c_ (and hence best statistical support). Zero values (in bold) therefore indicate the best-supported model. For the majority of individuals, the best supported model includes infectious (free) virus.
(PDF)

S5 Table Comparison of alternative adjusted cell-cell transmission kinetics using AIC_c_. Each row represents a different individual and columns represent different model structures. The original model (with infectious virus) is on the left, and the adjusted cell-cell transmission model (with different numbers of cell neighbors) is on the right. For each individual, numerical values indicate the difference in AIC_c_ between each model and the model with the lowest AIC_c_ (and hence best statistical support). Zero values (in bold) therefore indicate the best-supported model. For all individuals, the best supported model includes infectious (free) virus.
(PDF)
