# Supplementary figures and images for: Modeling the measles paradox reveals the importance of cellular immunity in regulating viral clearance
Source: PLoS Pathog. 2018 Dec 28;14(12):e1007493. doi: 10.1371/journal.ppat.1007493 (PMC6310241; doi:10.1371/journal.ppat.1007493)

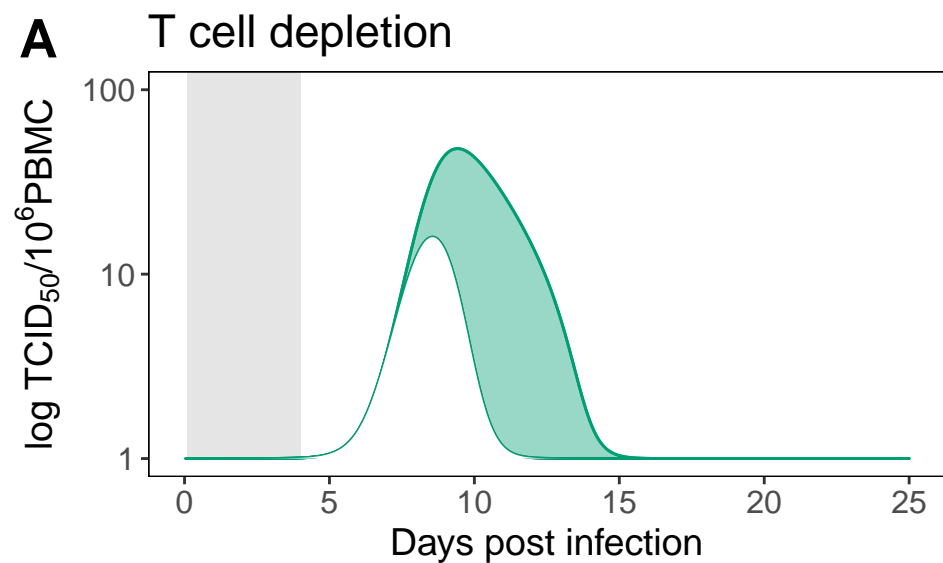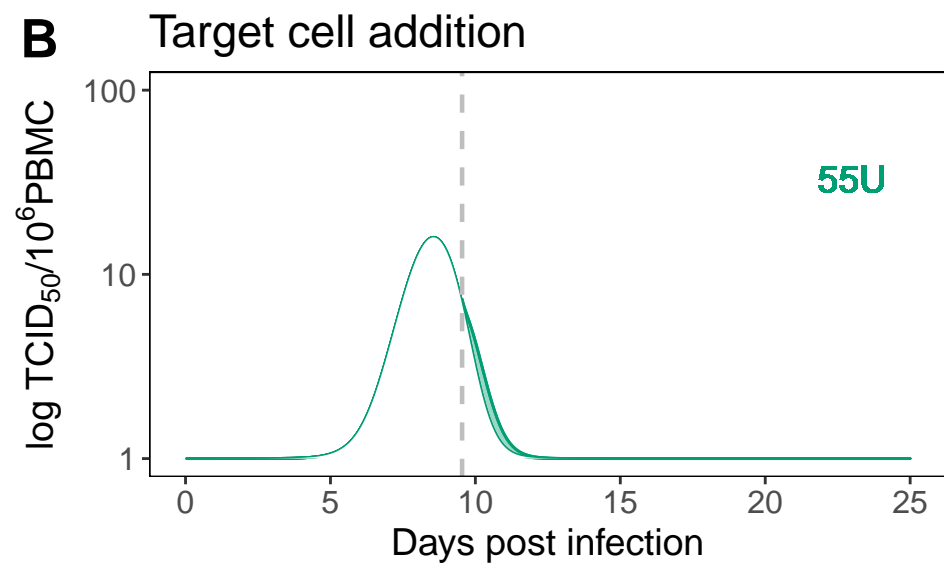

Supplement: S1 Fig — In each panel, upper green lines indicate the treatment effect ((A) T cell depletion or (B) target cell addition) and lower green lines indicate the corresponding control simulation. The effect of each treatment is calculated as the difference in the area under the curve (AUC) between the treatment and control simulations (green shaded regions), normalized by the AUC of the control simulation (white regions). The grey rectangular region represents the period of T cell depletion and the dashed grey vertical line represents the timing of target cell addition. (PDF) [file ppat.1007493.s001.pdf]

# Lymphocyte proliferation dynamics

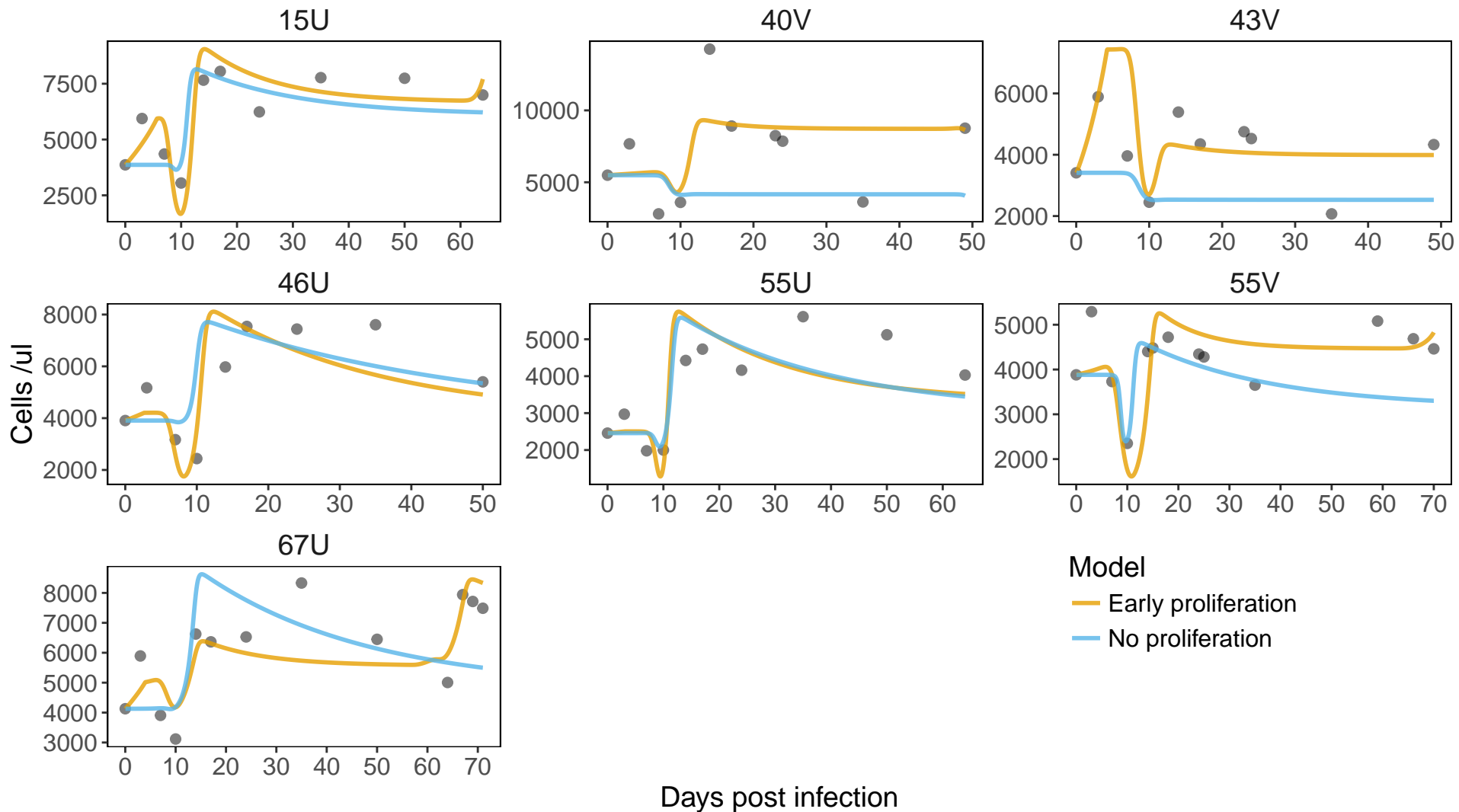

Supplement: S4 Fig — Solid lines indicate lymphocyte dynamics predicted by the target cell and T cell model without lymphocyte proliferation (blue) and with early lymphocyte proliferation (orange); points indicate lymphocyte data from Lin et al. (2012). Each panel corresponds to an individual macaque (indicated by the panel label). (PDF) [file ppat.1007493.s004.pdf]

55V

 $A_0$ 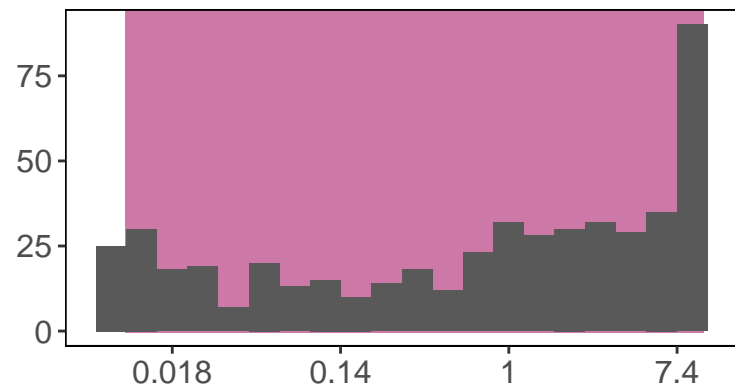 $\beta$ 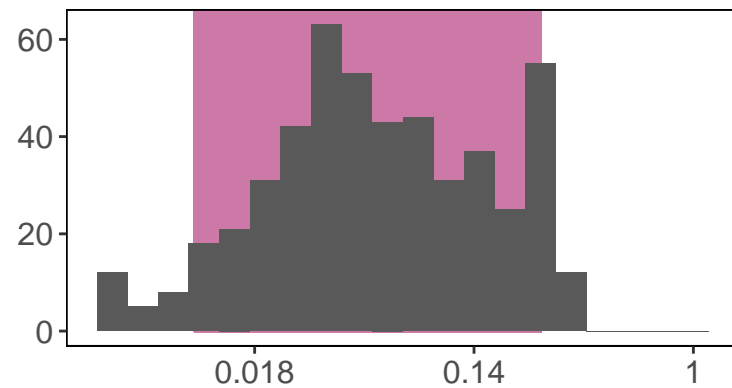 $k$ 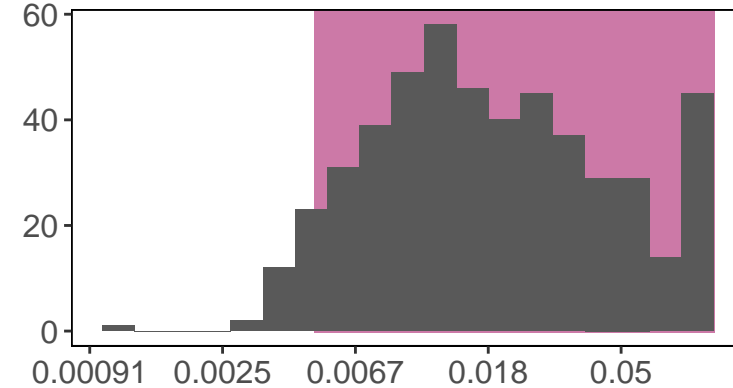 $p$ 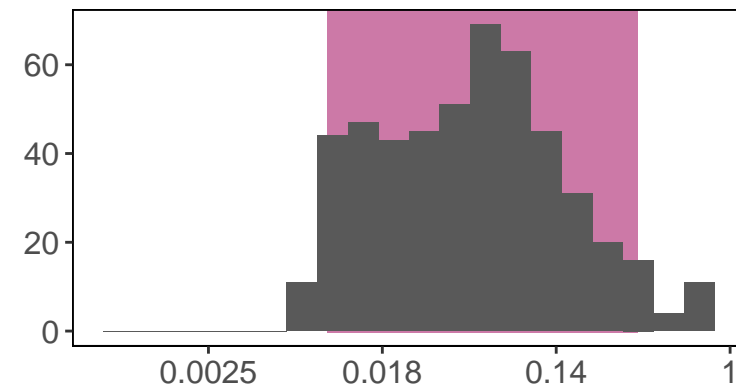 $q$ 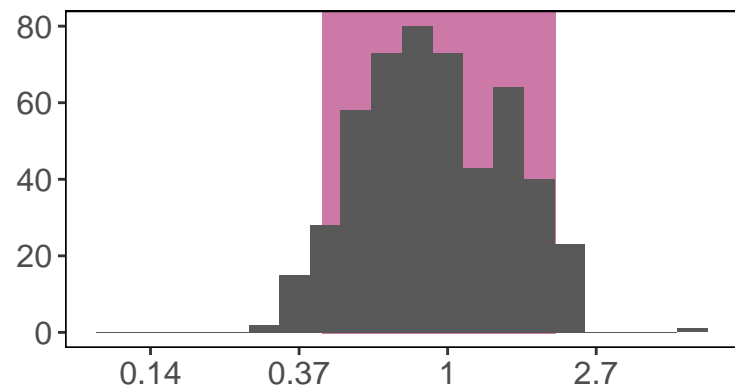 $q_s$ 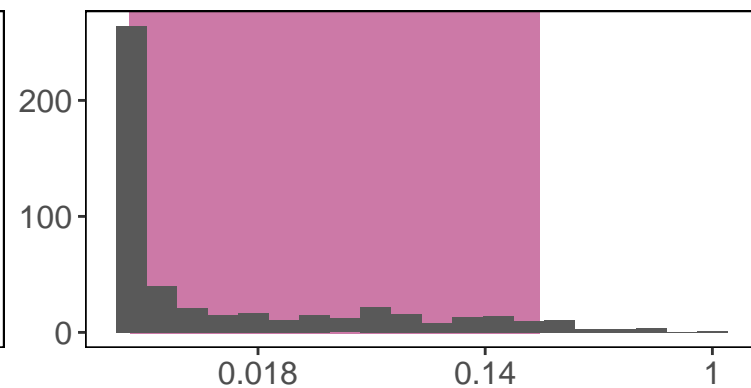 $r$ 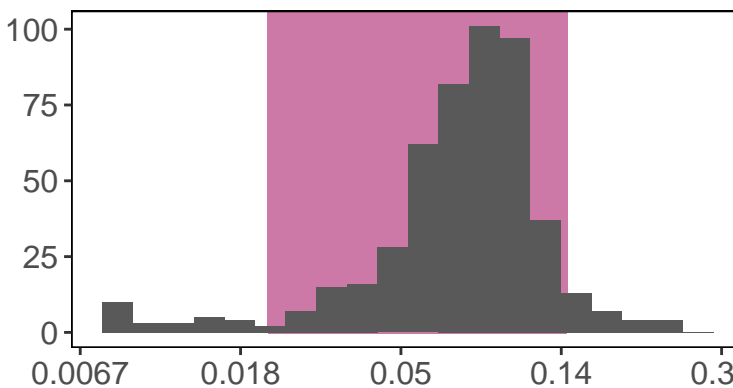 $R_0$ 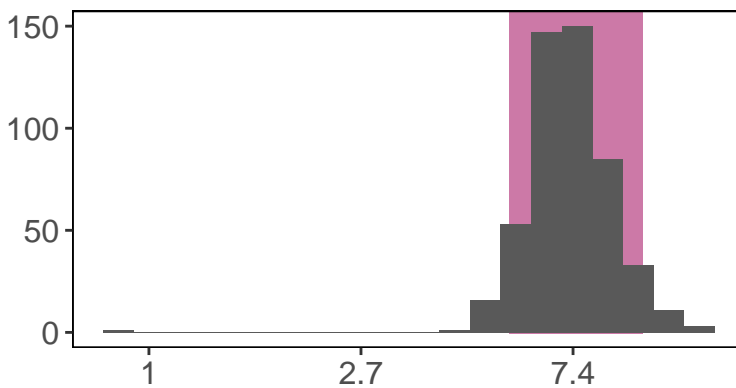 $R_0^*$ 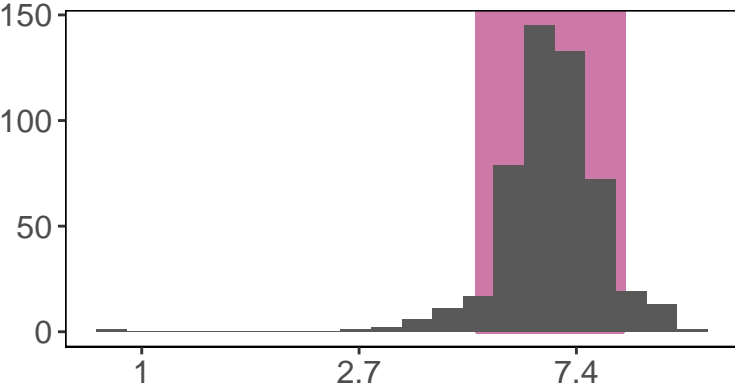 $s$ 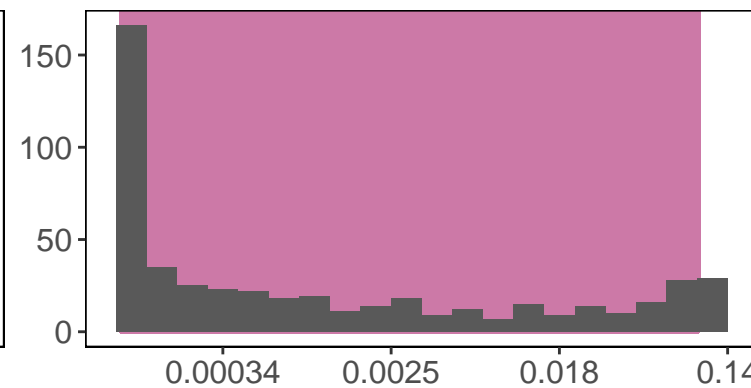 $t_d$ 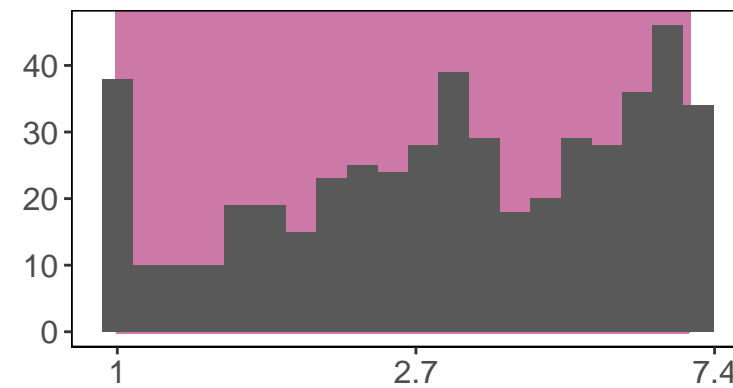 $V_0$ 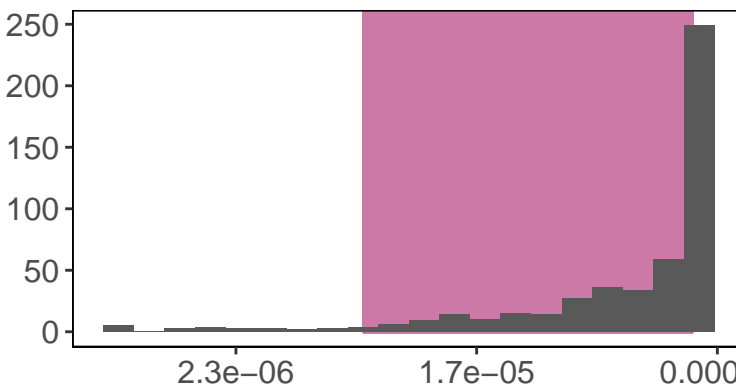

Parameter value

Supplement: S5 Fig — Histograms show fitted parameter estimates obtained from 500 bootstrap samples. R0 was calculated from the fitted parameters as pβS0/cδ; similarly R0* was calculated as pβ(S0 + A0)/c(δ + kA0). Shaded regions encompass the 90th percentiles. (PDF) [file ppat.1007493.s005.pdf]

Total viral load

1000  
100  
10  
1

15U

46U

55U

67U

40V

43V

55V

Individual

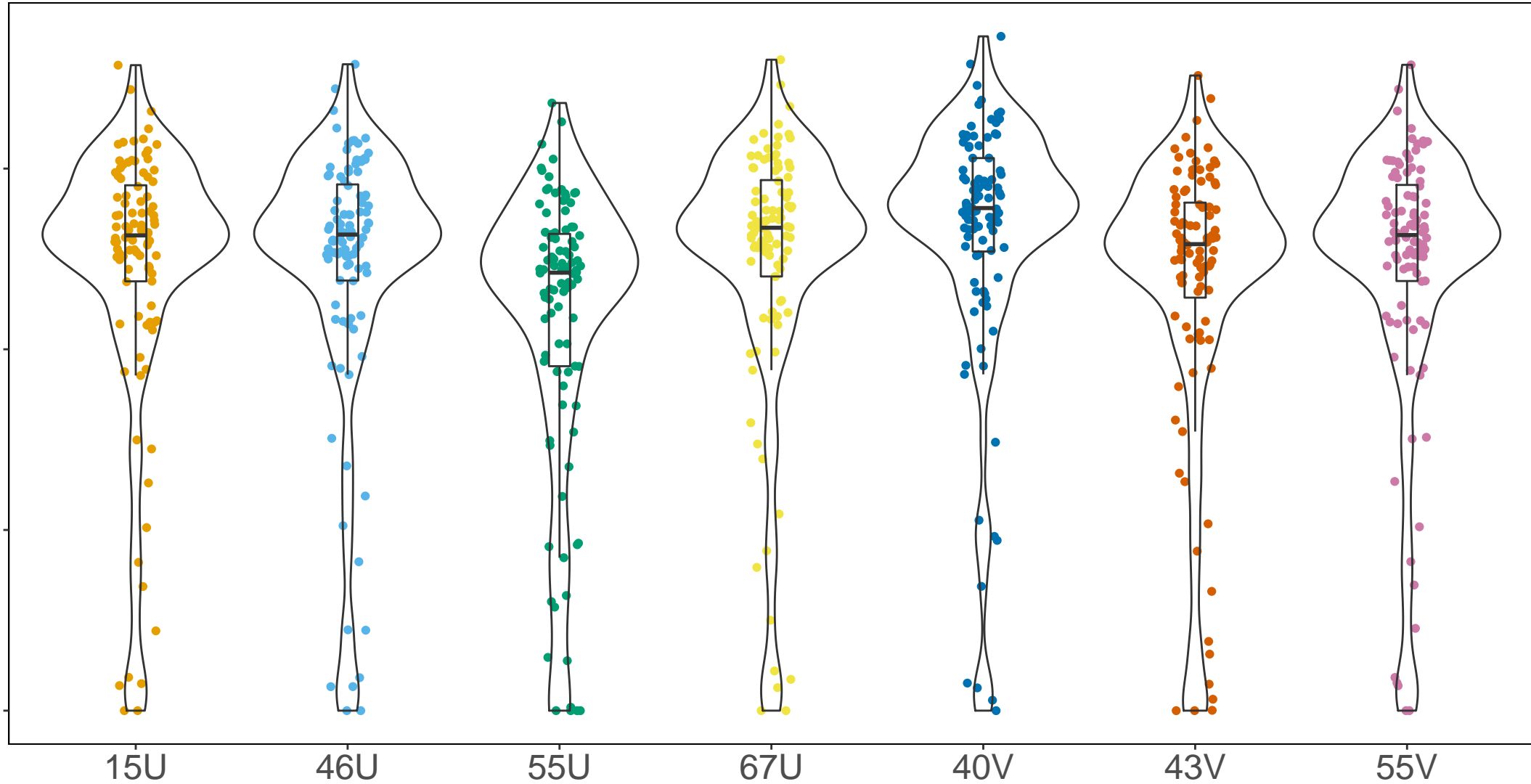

Supplement: S7 Fig — Each point represents the output (summarized here as total viral load) obtained from 1 of 100 different parameter sets generated by Latin Hypercube sampling. The corresponding distributions and box plots for each individual are outlined in black. (PDF) [file ppat.1007493.s007.pdf]

Partial rank correlation coefficient

15U

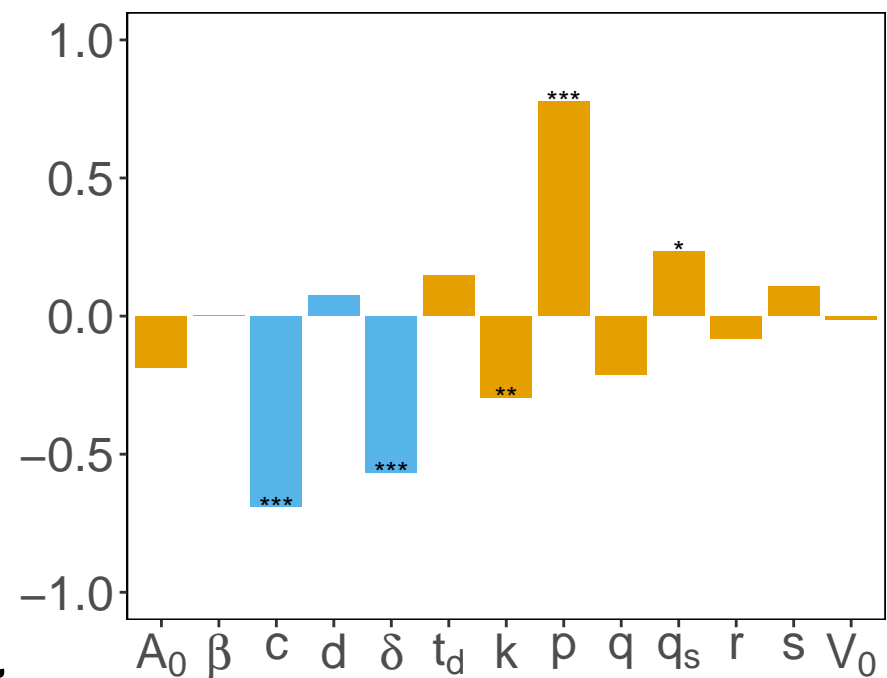

40V

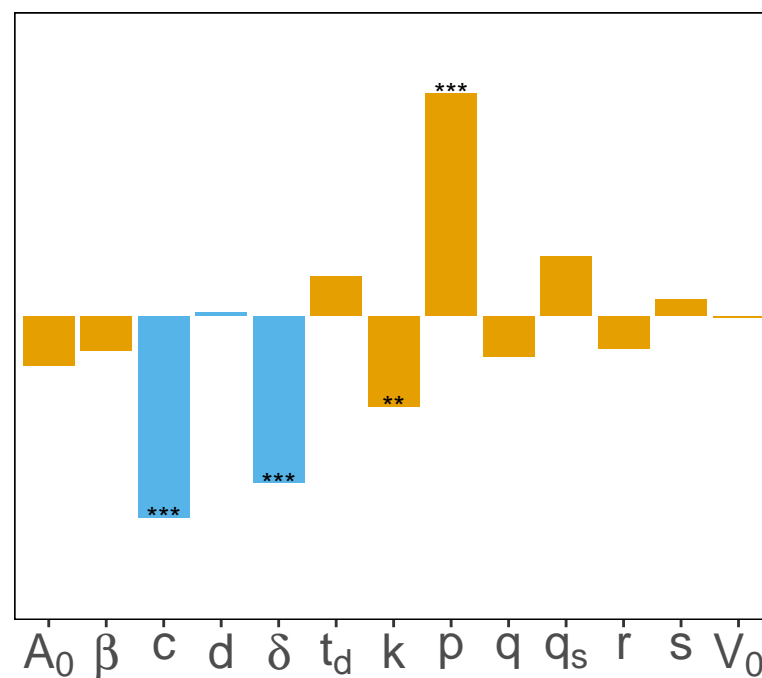

43V

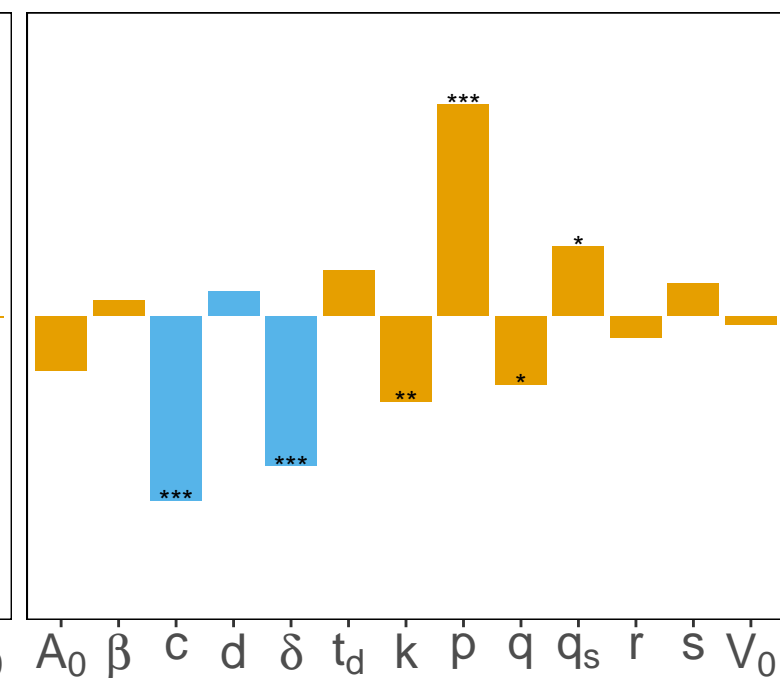

46U

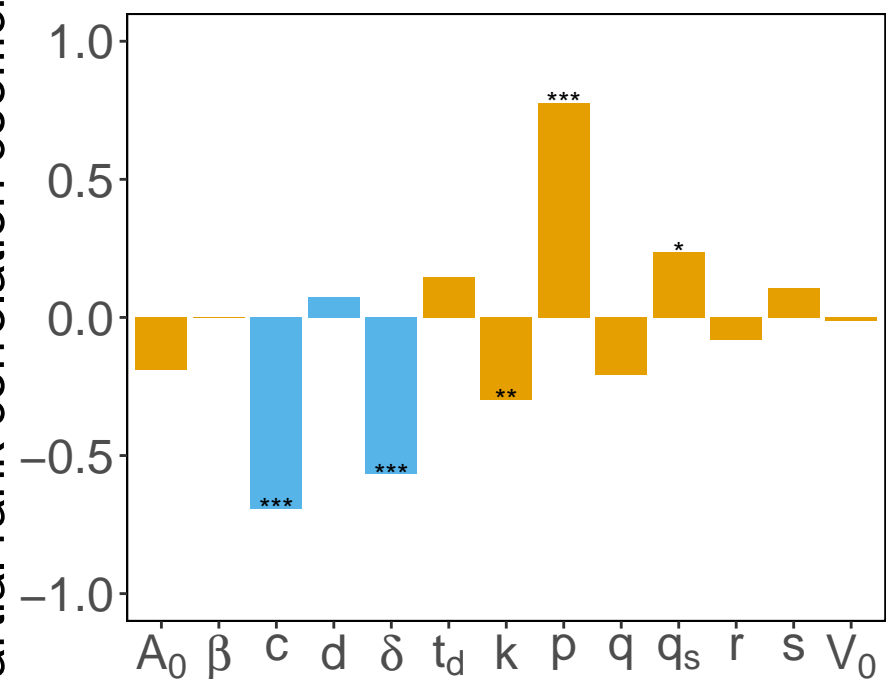

55U

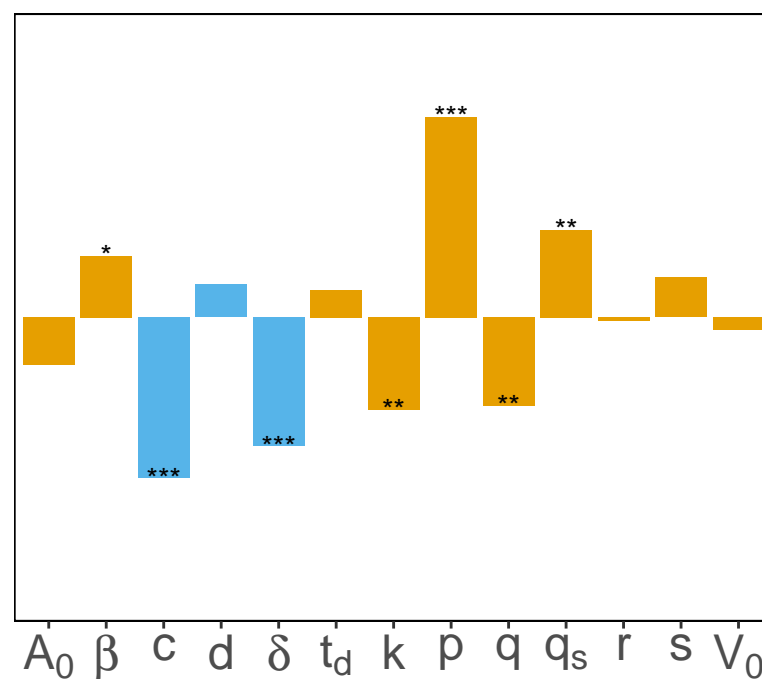

55V

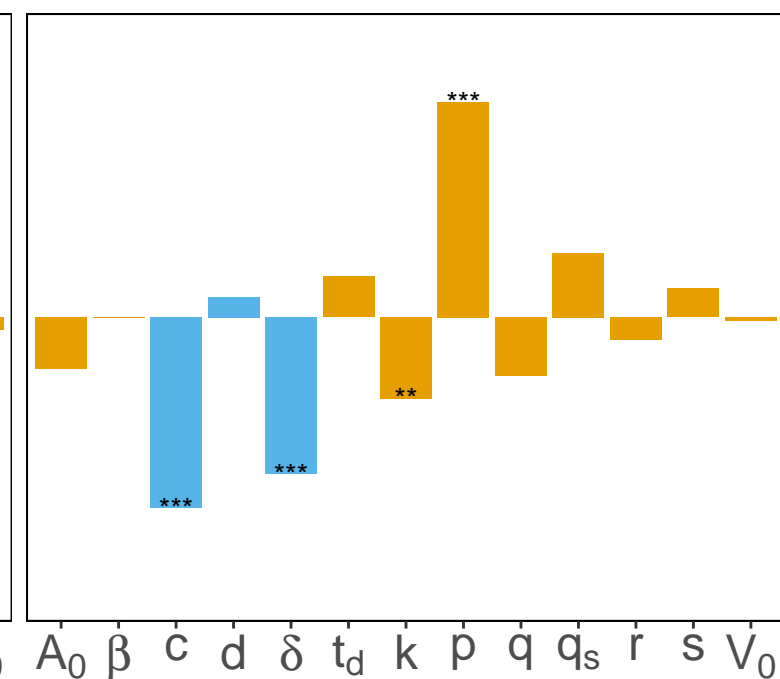

67U

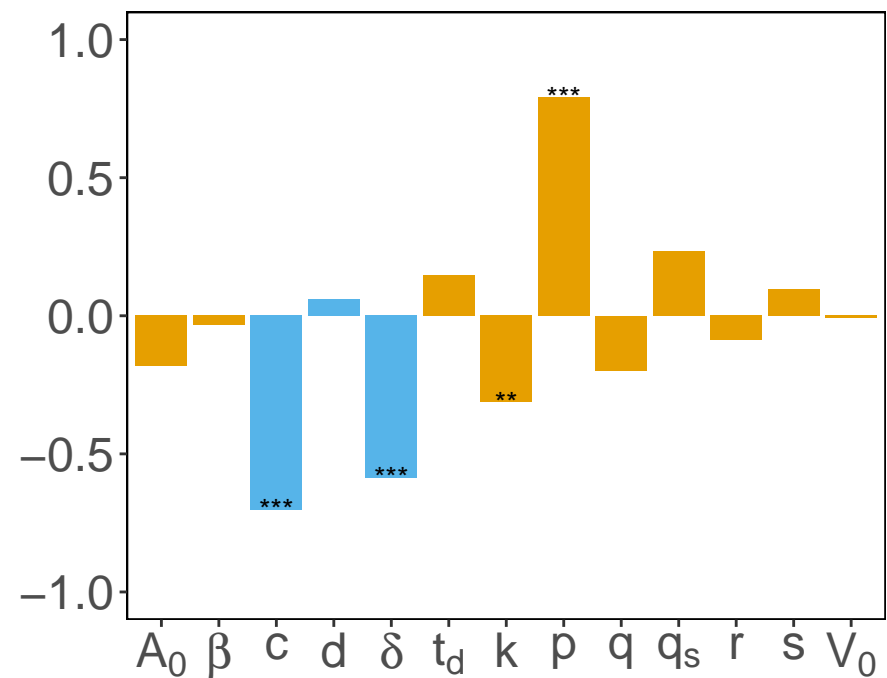

estimated  
fixed

Parameter

Supplement: S8 Fig — Each bar represents a different parameter, and the absolute height represents the magnitude of model sensitivity to that parameter. Positive values indicate that an increase in parameter value causes a positive change in the measured model output (i.e. an increase in total viral load), whereas negative values indicate a negative change. Note that the scaling factor, ψ, was omitted from this analysis as it does not appear in the model equations or directly impact the resulting predictions. Each panel corresponds to an individual macaque (identified by the panel label). Significance thresholds are defined as follows: *p < 0.05, **p < 0.01, ***p < 0.001. (PDF) [file ppat.1007493.s008.pdf]

**A** Initial # T cells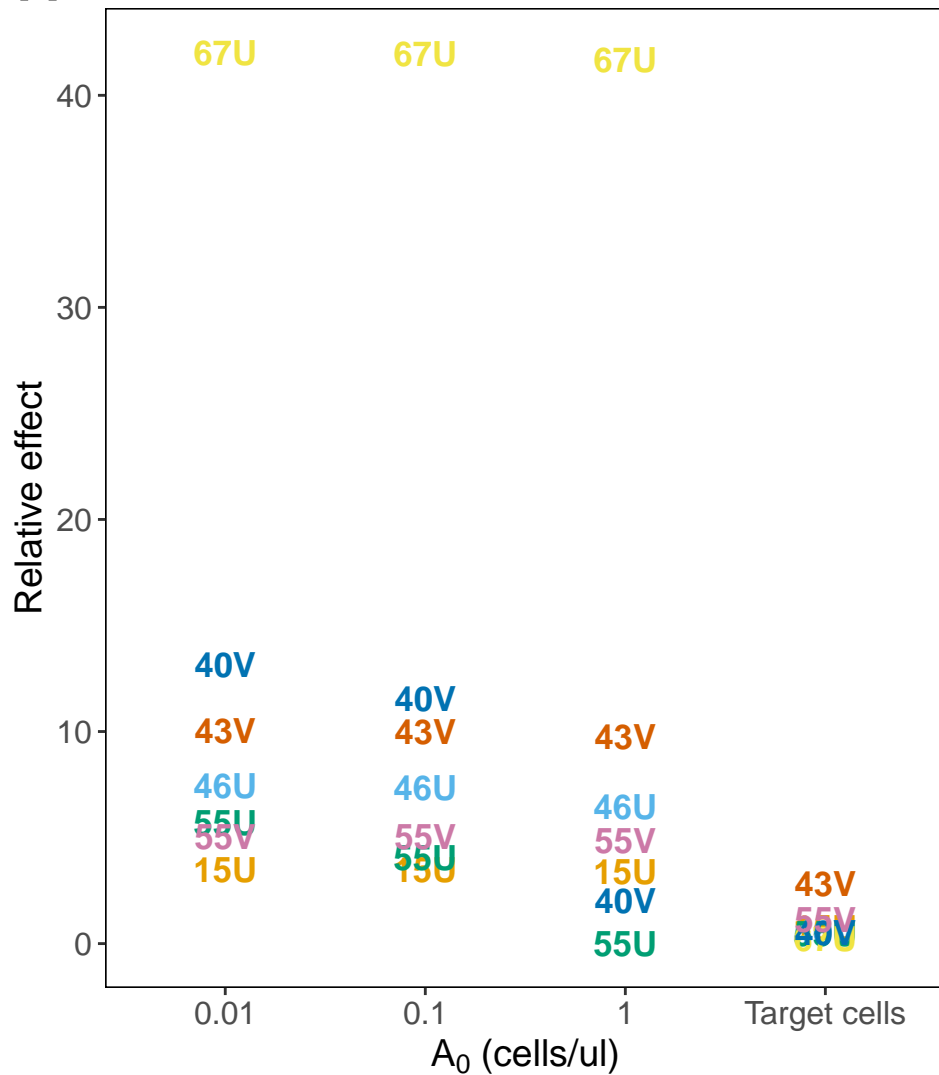**B** Duration of suppression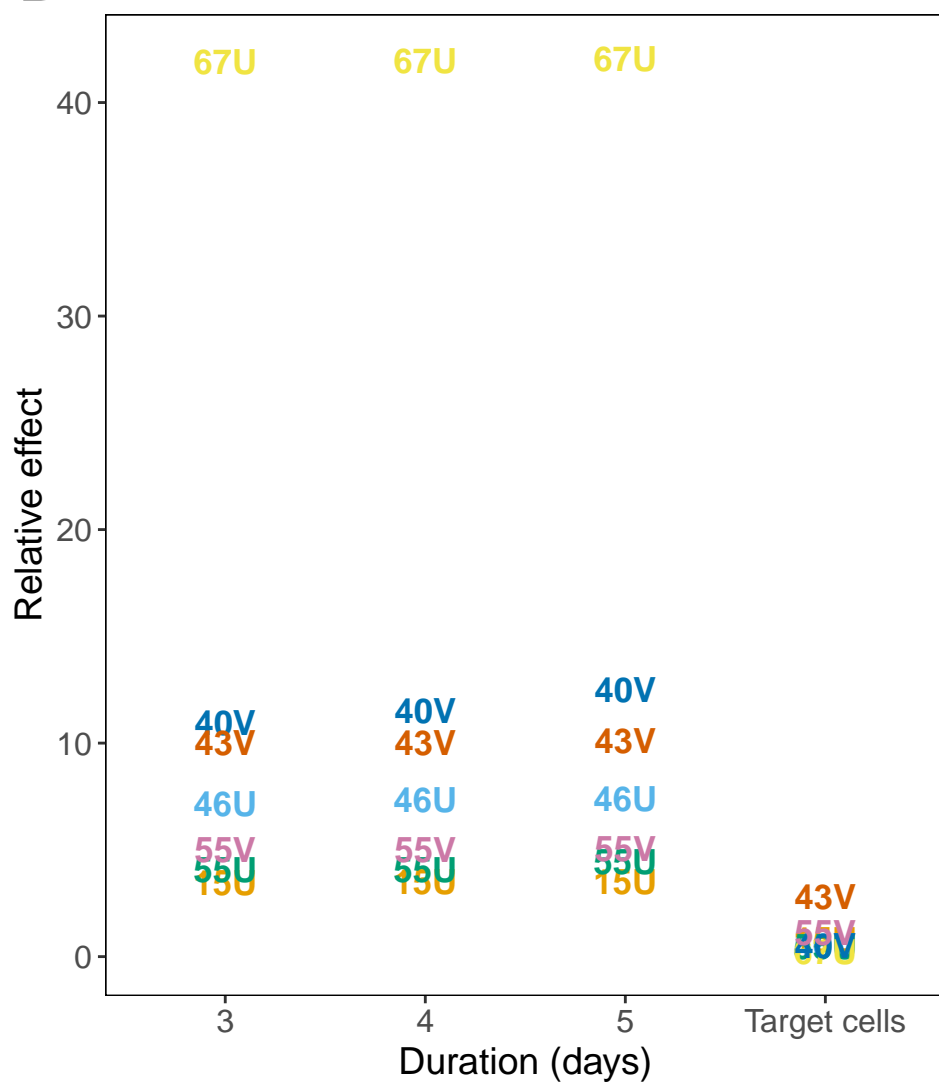

Supplement: S9 Fig — The relative change in viral load (or ‘relative effect’) was recalculated whilst: (A) the initial number of activated T cells (A0) was varied between 0.01, 0.1 (original condition), and 1 cells/μl; and (B) the duration of T cell suppression was varied between 3, 4 (original condition), and 5 days. On each panel, the effect of the original target cell addition experiment (‘Target cells’) is shown for comparison. For each macaque, the relative change in viral load was calculated as the difference in the area under the curve (AUC) between the experimental and control simulations, normalized by the AUC of the control simulation. Results for each individual are indicated by the corresponding identification code. In all but one case (individual 55U when A0 = 1 cells/μl), the effect of T cell depletion for each individual remains greater than that of target cell addition. (PDF) [file ppat.1007493.s009.pdf]

**A** Magnitude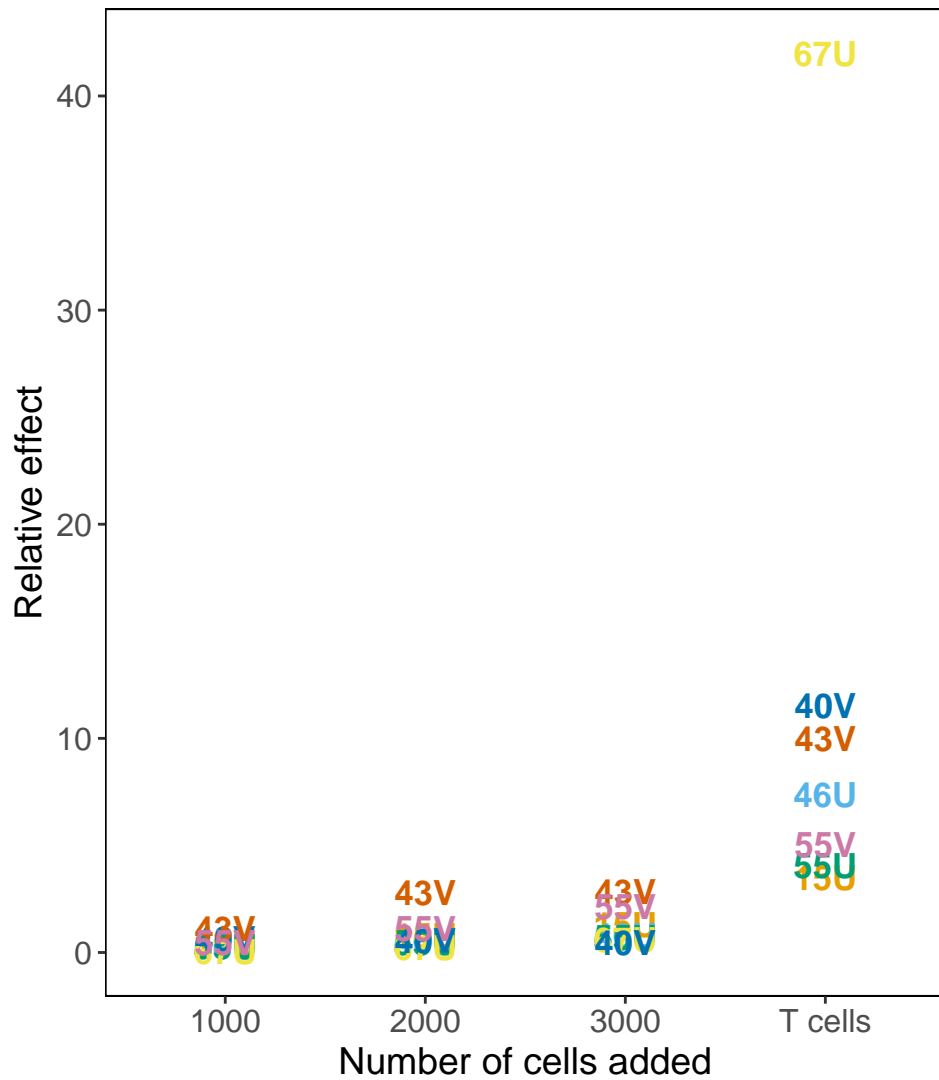**B** Timing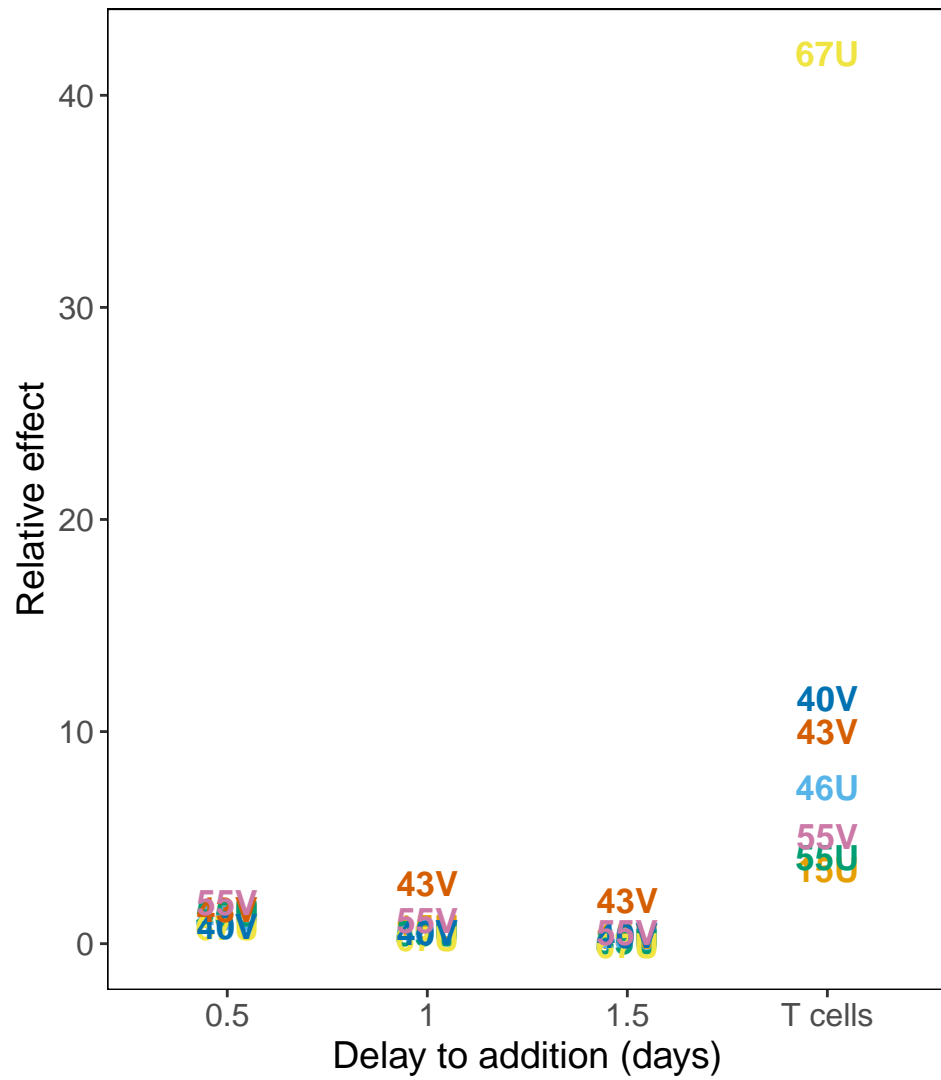

Supplement: S10 Fig — The relative change in viral load (or ‘relative effect’) was recalculated whilst: (A) the number of added target cells was varied between 1000, 2000 (original condition), and 3000 cells/μl; and (B) the timing of target cell addition was varied between 0.5, 1 (original condition), and 1.5 days following peak viral load. On each panel, the effect of the original T cell depletion experiment (‘T cells’) is shown for comparison. For each macaque, the relative change in viral load was calculated as the difference in the area under the curve (AUC) between the experimental and control simulations, normalized by the AUC of the control simulation. Results for each individual are indicated by the corresponding identification code. In all cases the effect of target cell addition for each individual remains less than that of T cell depletion. (PDF) [file ppat.1007493.s010.pdf]

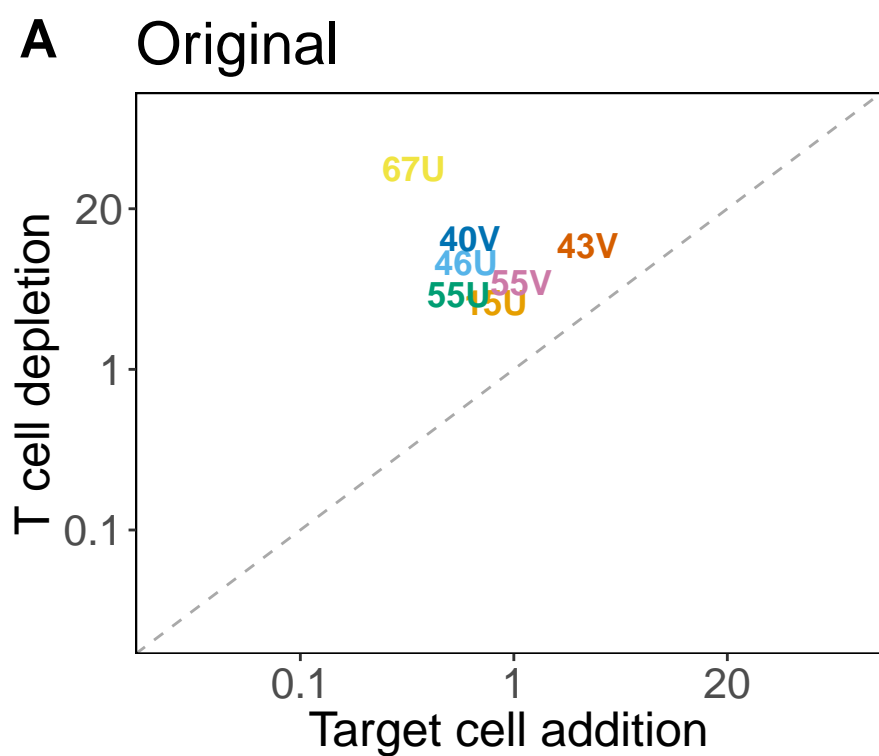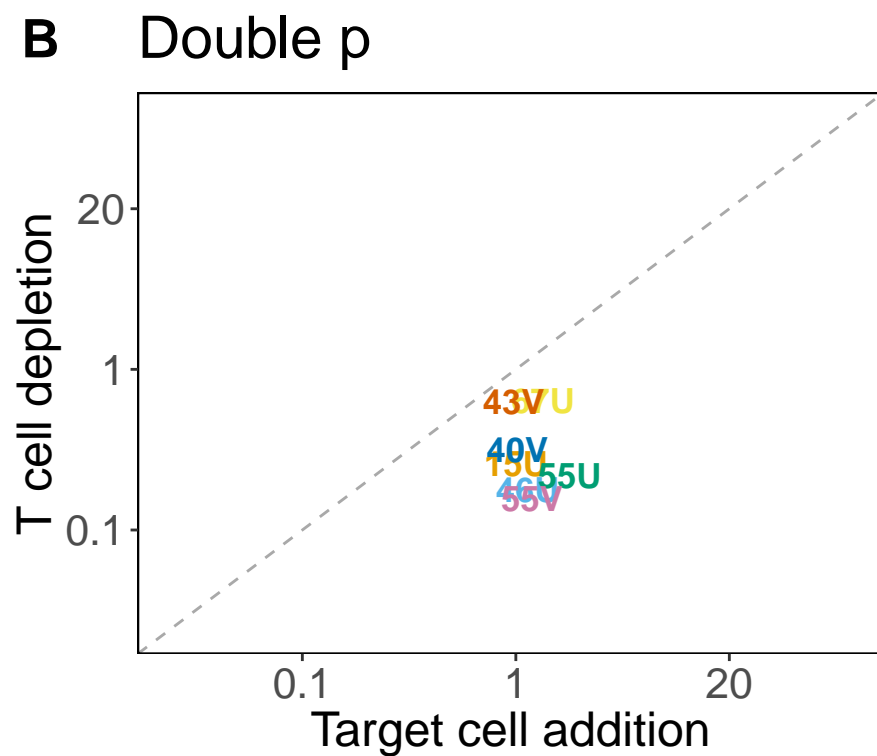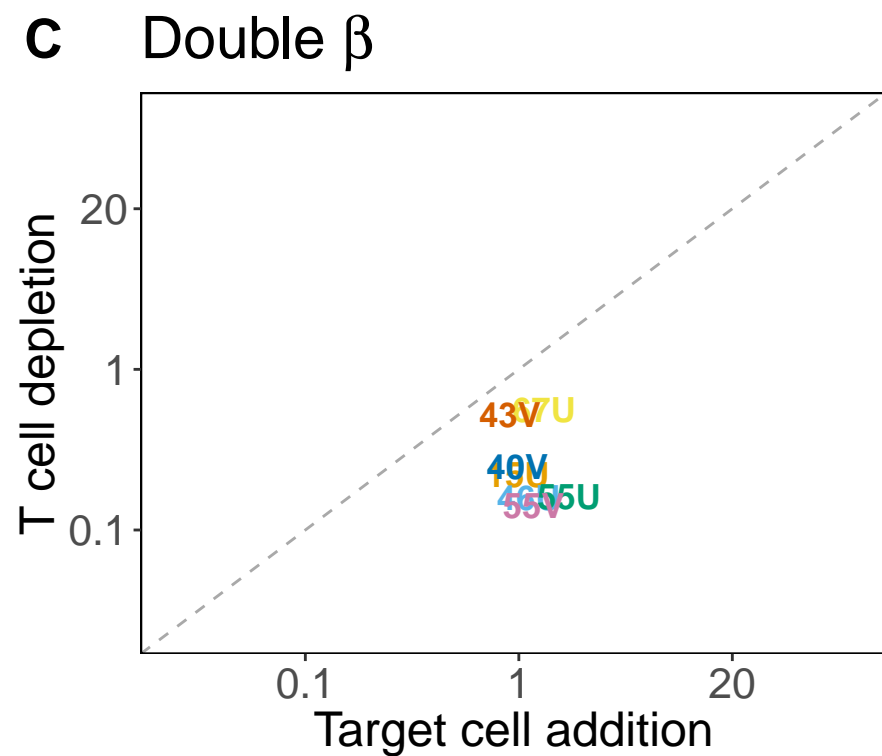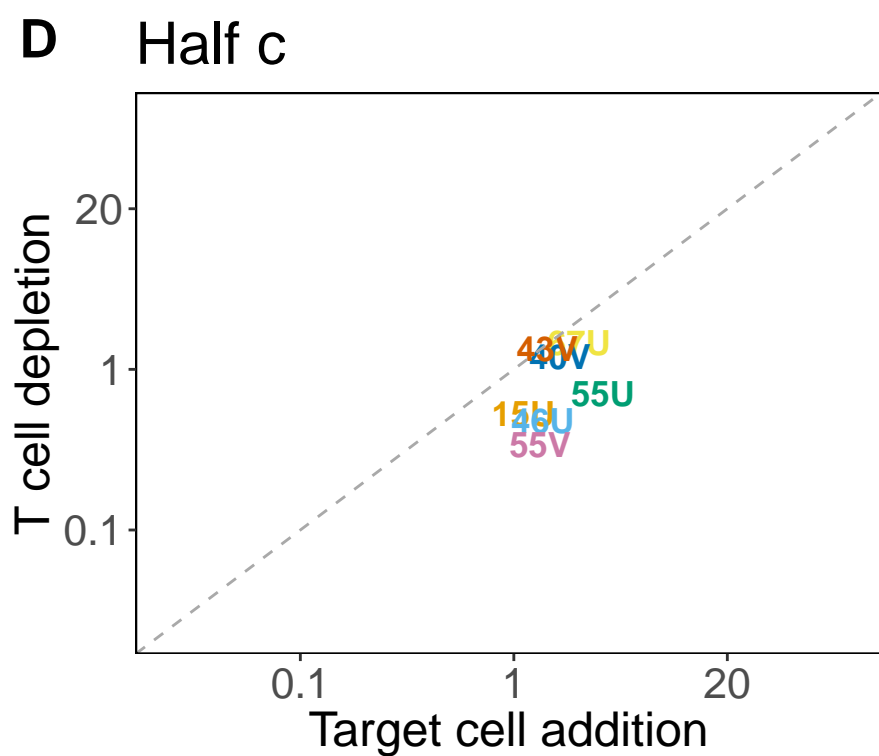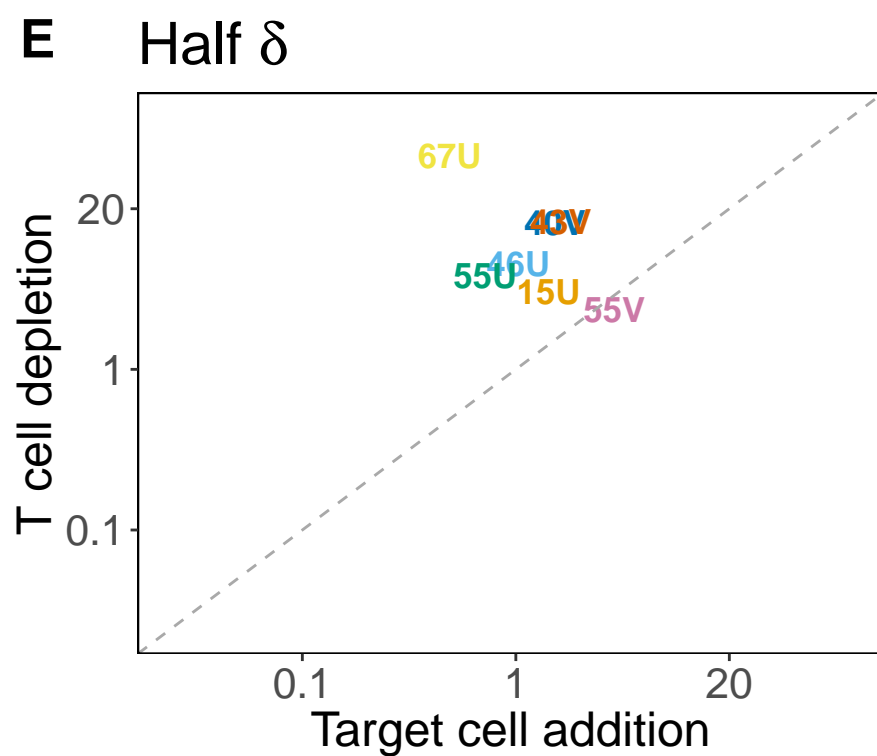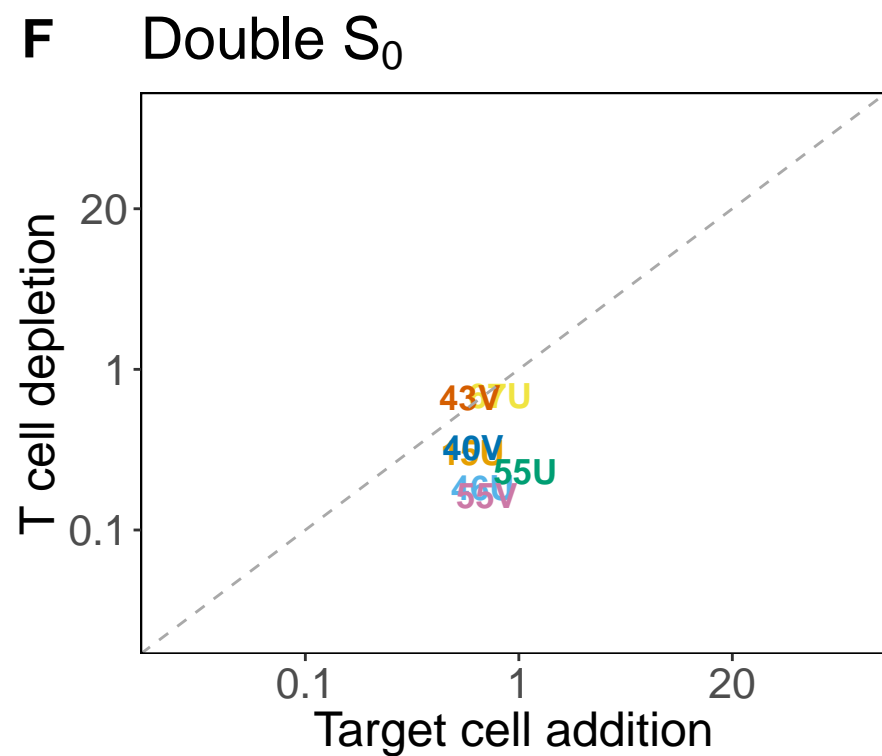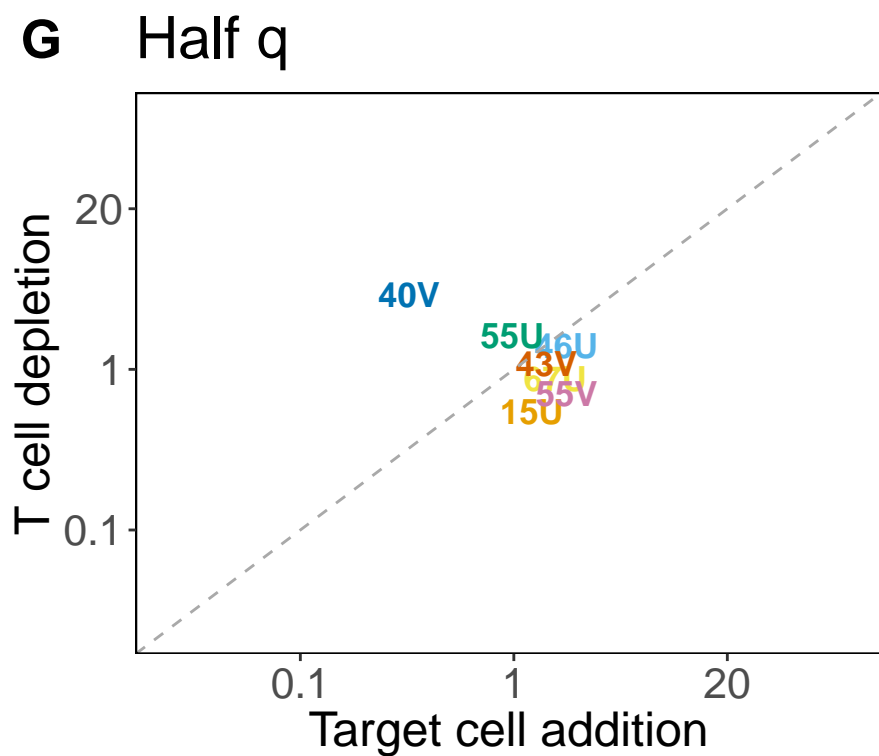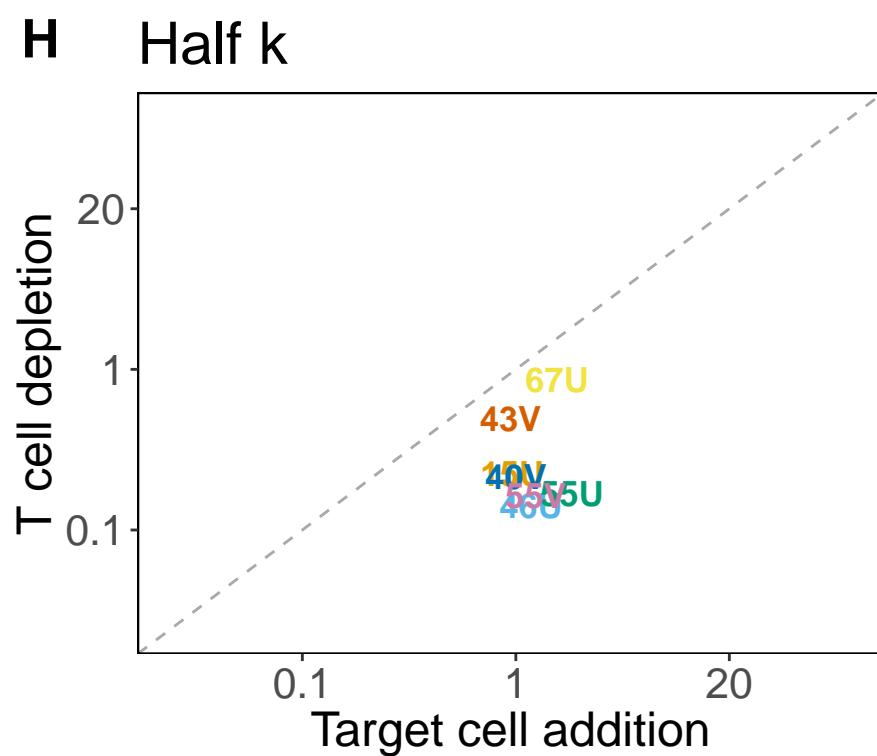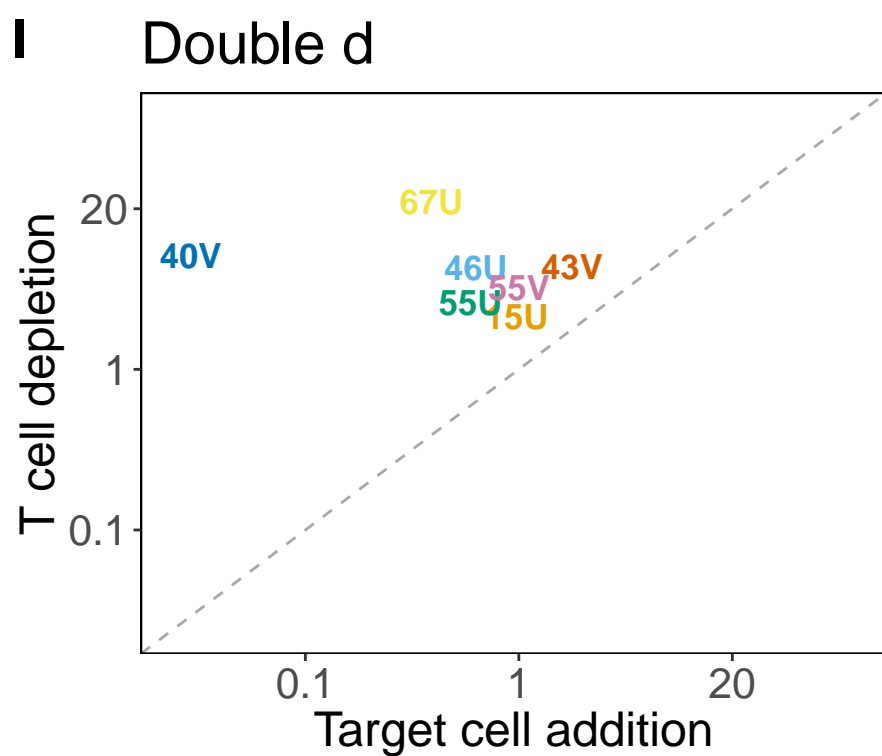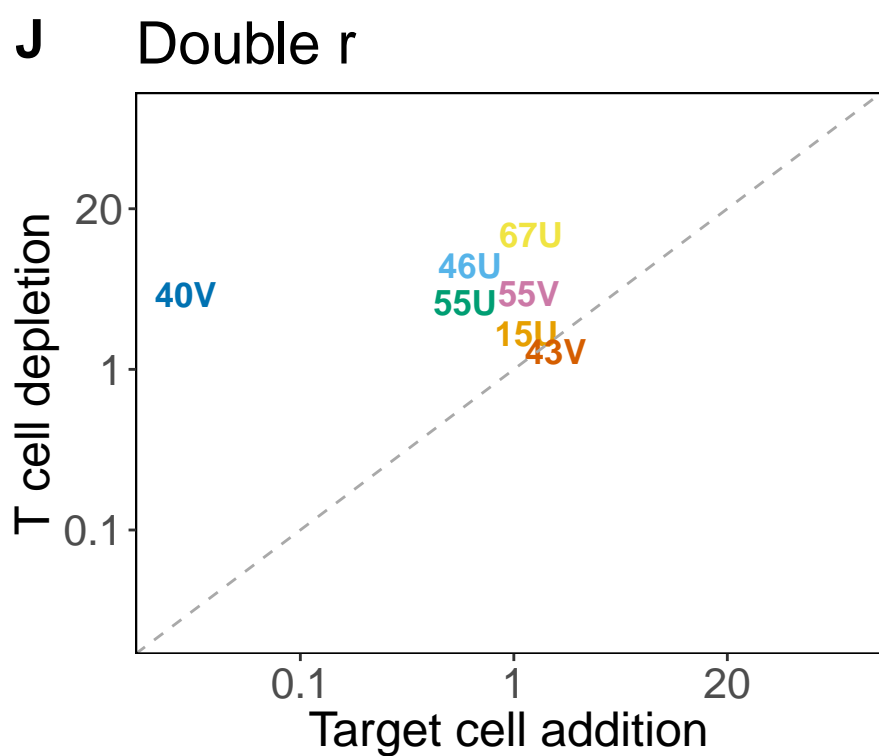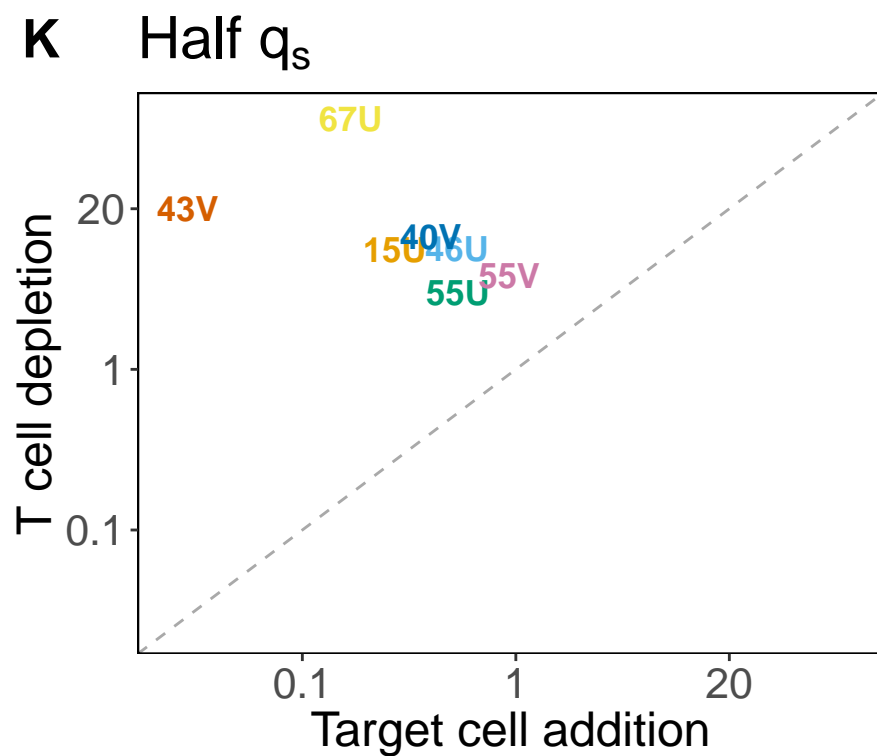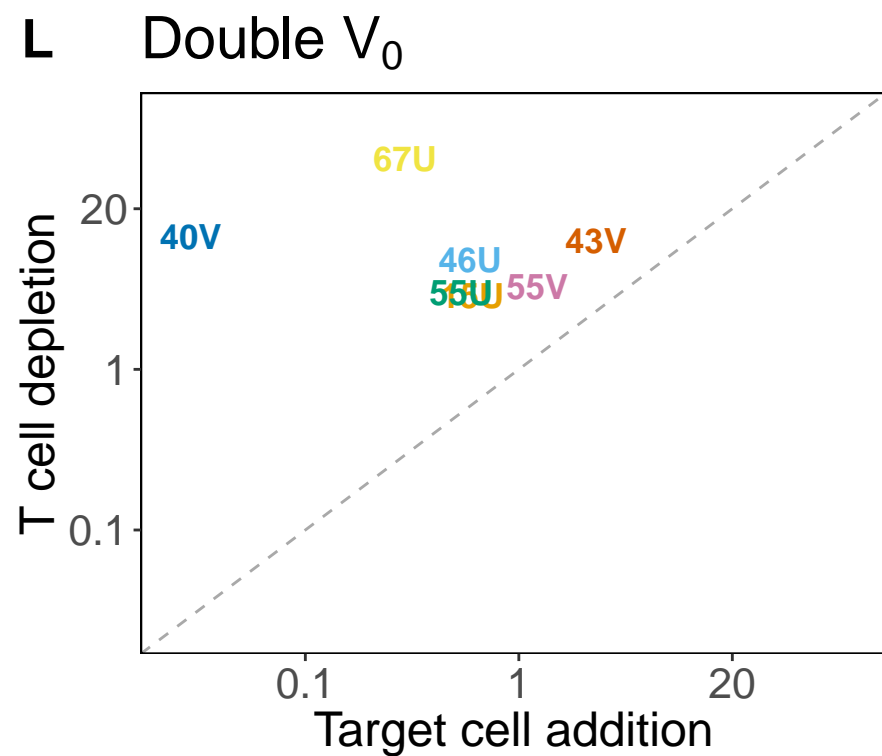

Supplement: S11 Fig — For each macaque, the relative effect of each experiment is calculated as the difference in the area under the viral load curve (AUC) between the experimental and control simulations, normalized by the AUC of the control simulation. Results for each individual are indicated by the corresponding identification code. Parameters from the original target cell and T cell model are given in panel A. All other panels represent the impact of changing one parameter (indicated by the panel title). Panels (B)–(F) represent changes that cause R0 = pβS0/cδ, the within-host viral fitness, to double. Panels (G)–(I) represent changes that reduce the strength of the T cell response. Panels (J)–(L) represent other parameter changes that are not directly related to viral fitness or T cell predation. (PDF) [file ppat.1007493.s011.pdf]

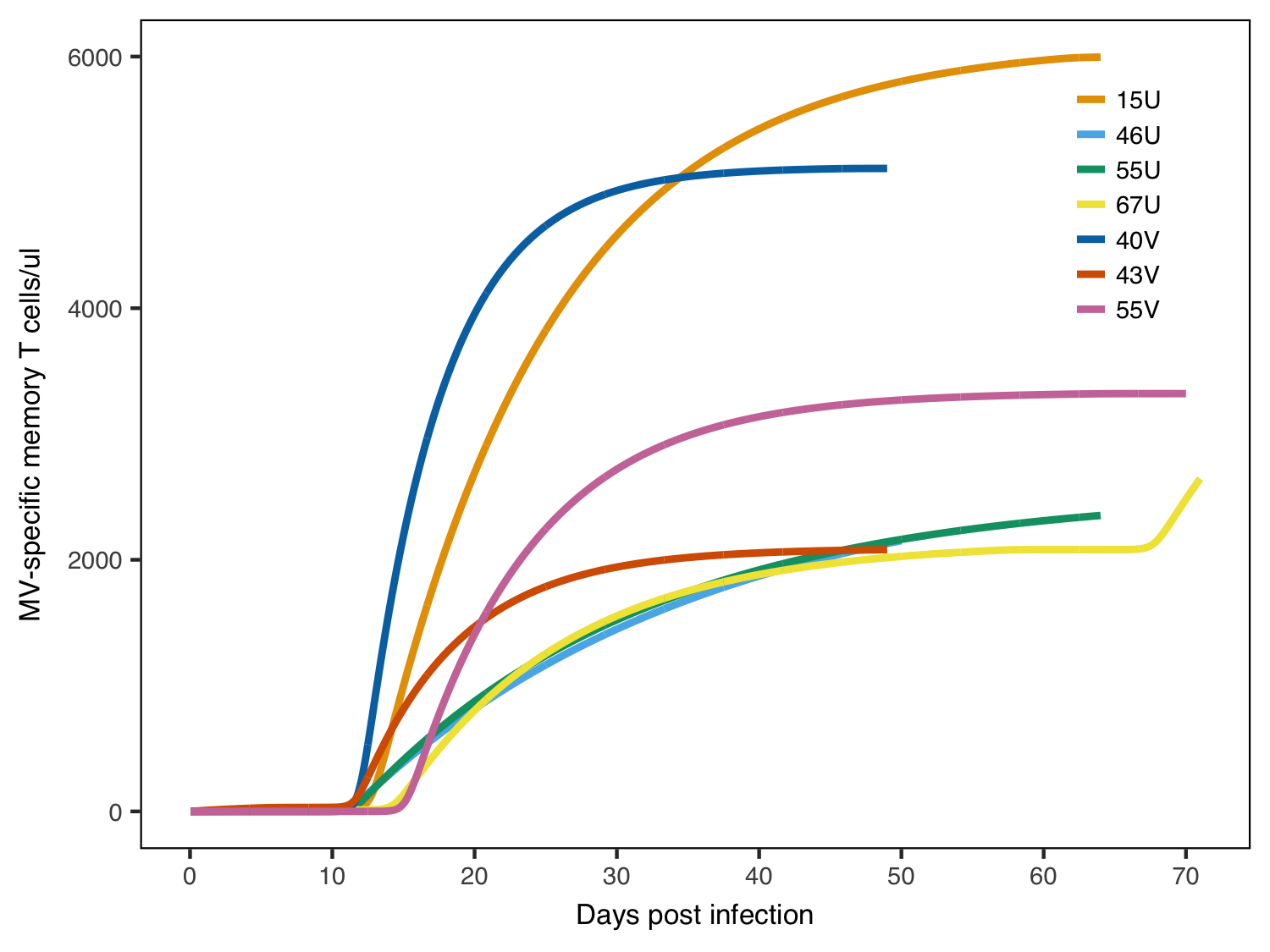

Supplement: S12 Fig — For each macaque, the development of MV-specific memory T cells can be predicted by tracking the activated T cells that transition to the susceptible cell compartment. Solid lines indicate the predictions of the best-fitting target cell and T cell model, and each color represents an individual macaque (with identification codes in the inset legend). (TIF) [file ppat.1007493.s012.tif]

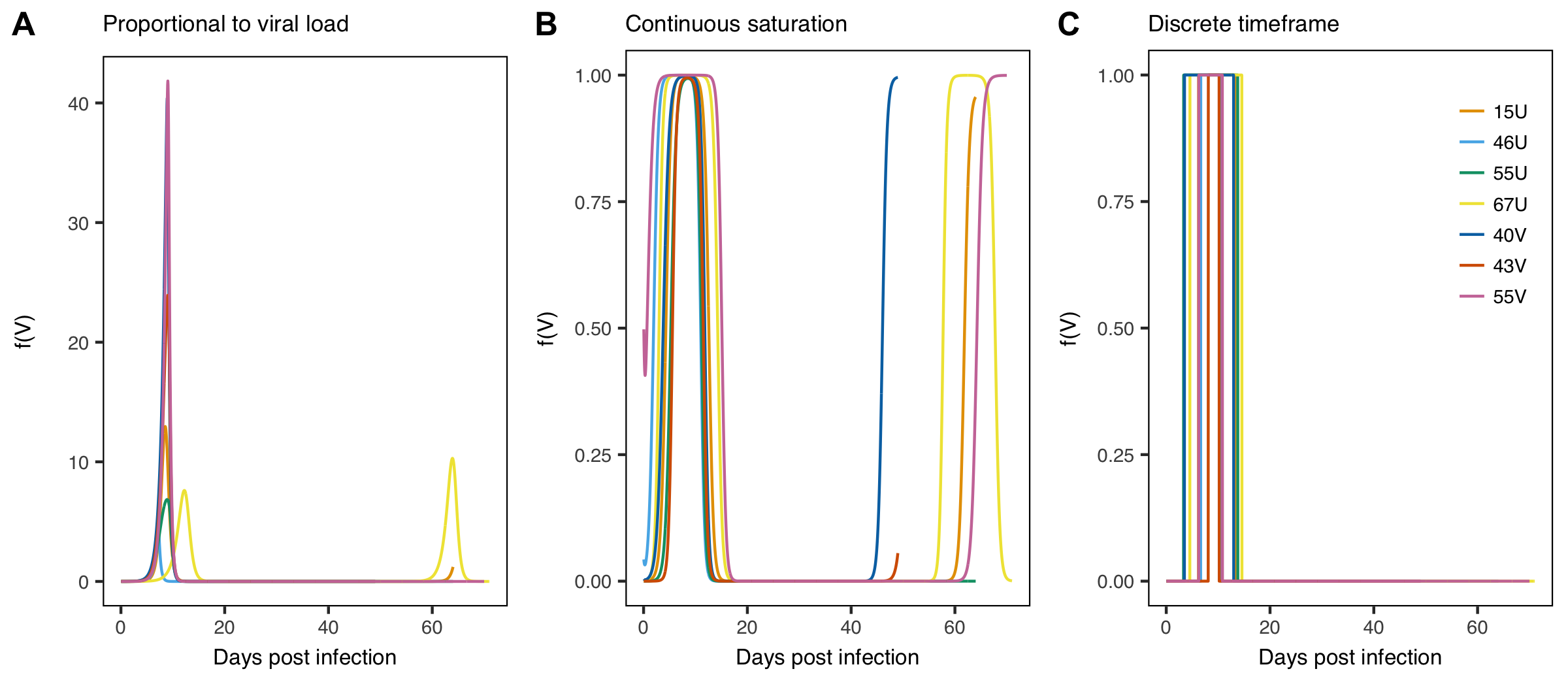

Supplement: S13 Fig — Three different functions are used to model the activation of MV-specific T cells, f(V): (A) activation that is proportional to viral load; (B) activation that saturates at high viral loads; and (C) constant activation within a discrete timeframe. Solid lines indicate f(V) for each model, and each color represents an individual macaque (with identification codes in panel C). Mathematical formulae for f(V) are given in the Materials and methods and S1 Appendix. (TIF) [file ppat.1007493.s013.tif]

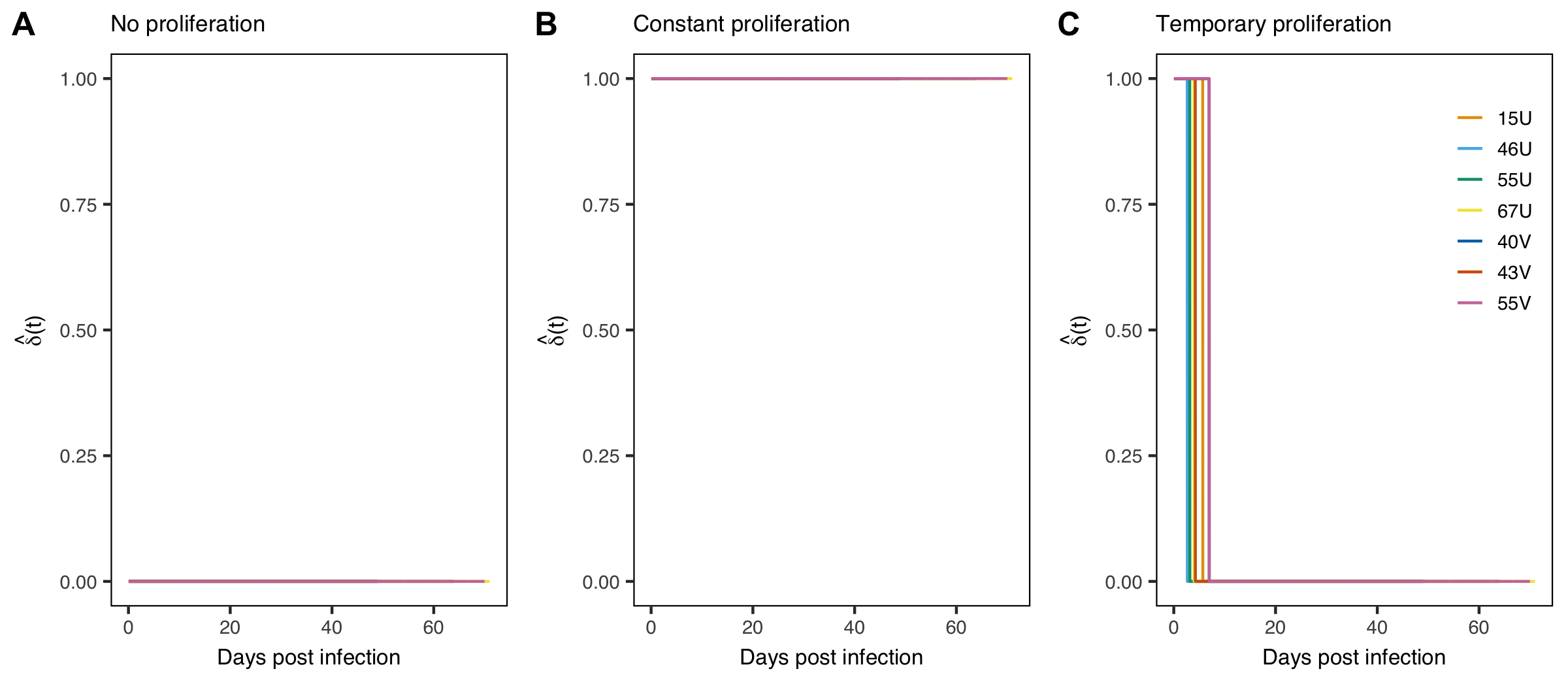

Supplement: S14 Fig — Three different functions are used to model the proliferation of susceptible lymphocytes, δ^(t): (A) no proliferation; (B) constant proliferation; and (C) constant proliferation within a temporary timeframe. Solid lines indicate δ^(t) for each model, and each color represents an individual macaque (with identification codes in panel C). Mathematical formulae for δ^(t) are given in the Materials and methods and S1 Appendix. (TIF) [file ppat.1007493.s014.tif]

**A**

No proliferation

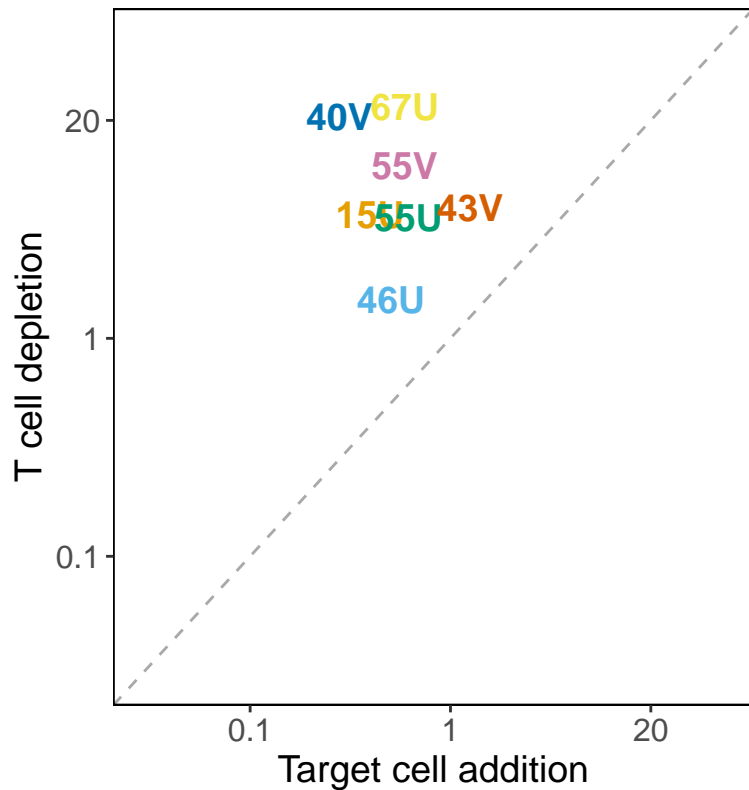**B**

Constant proliferation

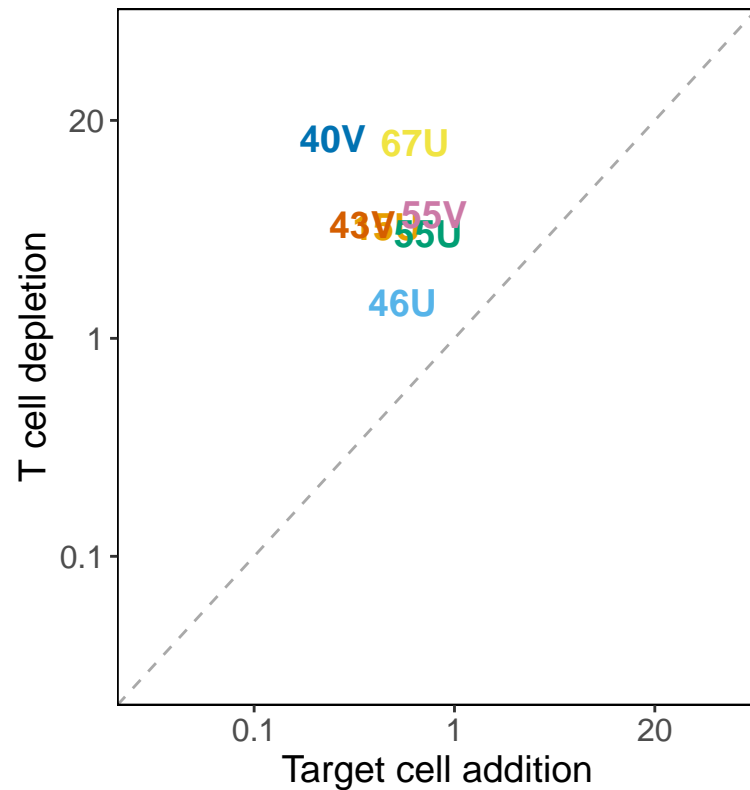**C**

Temporary proliferation

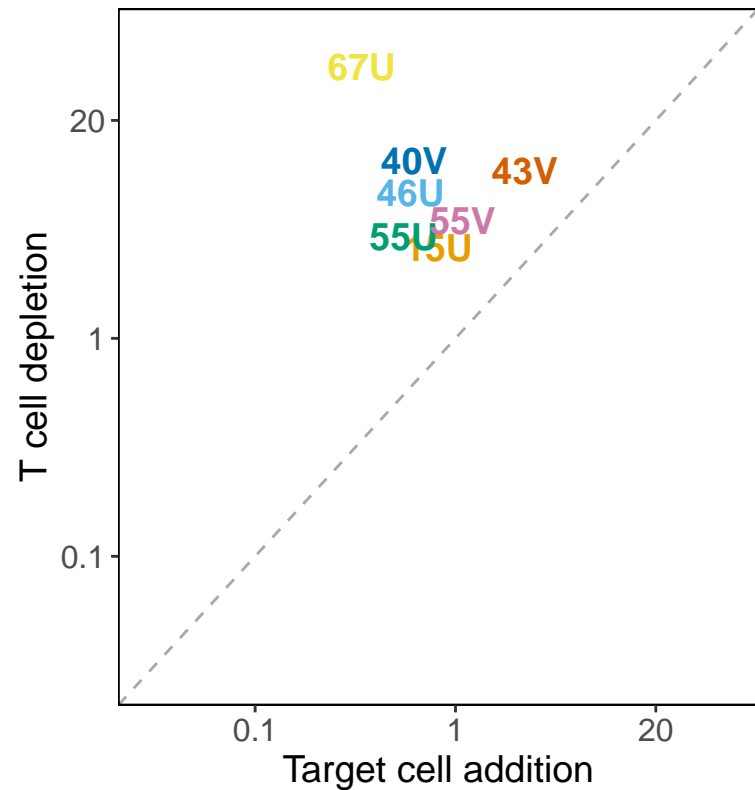

Supplement: S15 Fig — Three different functions are used to model the proliferation of susceptible lymphocytes, δ^(t): (A) no proliferation; (B) constant proliferation; and (C) constant proliferation within a temporary timeframe. For each macaque, the impacts of T cell depletion and target cell addition on viral load were calculated as the difference in area under curve (AUC) between the experimental and control simulations, normalized by the AUC of the control simulation. Results for each individual are indicated by the corresponding identification code and the dashed line signifies the y = x boundary where experimental effects are equal. Mathematical formulae for all proliferation functions are given in the Materials and methods and S1 Appendix. (PDF) [file ppat.1007493.s015.pdf]

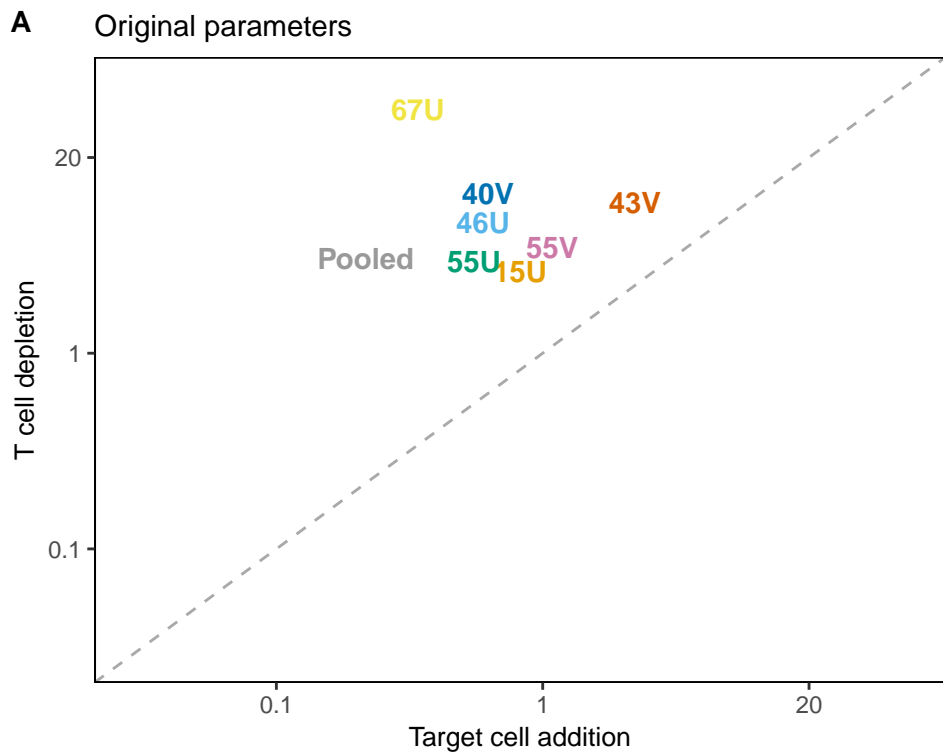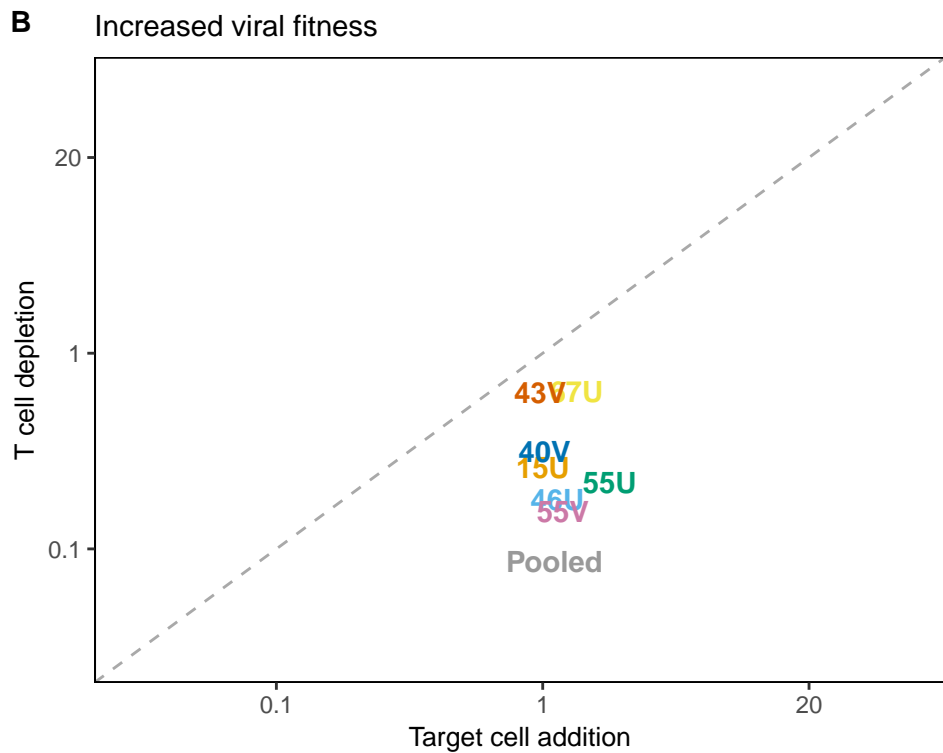

Supplement: S16 Fig — For each individual (or pooled) fit, the impacts of T cell depletion and target cell addition on viral load were calculated as the difference in area under curve (AUC) between the experimental and control simulations, normalized by the AUC of the control simulation. Results for each individual are indicated by the corresponding identification code and the dashed line signifies the y = x boundary where experimental effects are equal. Results for the pooled data are indicated by the grey ‘Pooled’ label. Simulations were conducted for (A) MV (by using best-fit parameters from the original target cell and T cell model); and (B) a virus with increased fitness (by doubling the viral replication rate, p, of each individual (or pooled) fit). (PDF) [file ppat.1007493.s016.pdf]

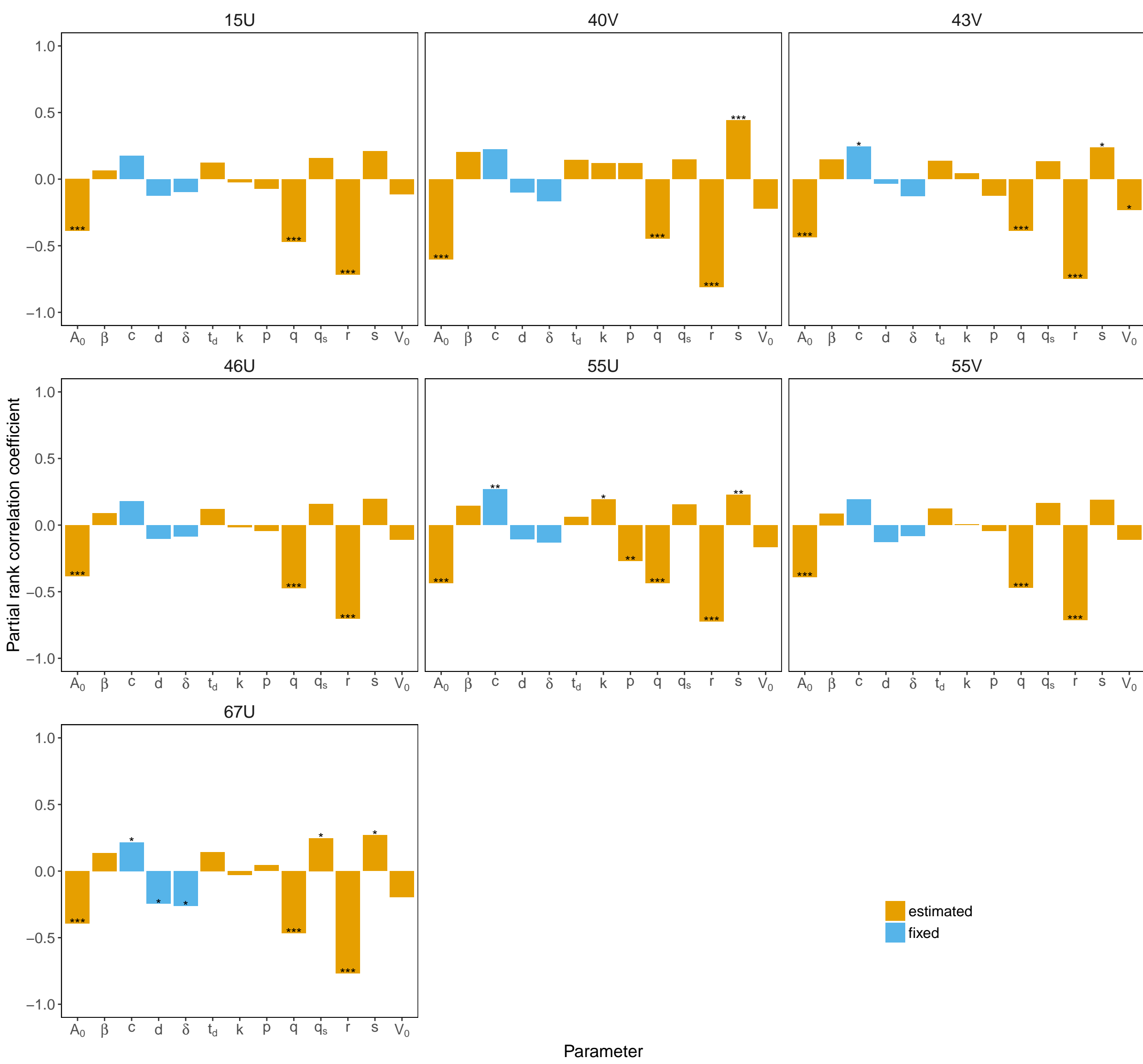

Supplement: S17 Fig — Each bar represents a different parameter, and the absolute height represents the magnitude of model sensitivity to that parameter. Positive values indicate that an increase in parameter value causes a positive change in the measured model output (i.e. the time to virus rebound), whereas negative values indicate a negative change. Note that the scaling factor, ψ, was omitted from this analysis as it does not appear in the model equations or directly impact the resulting predictions. Each panel corresponds to an individual macaque (identified by the panel label). Significance thresholds are defined as follows: *p < 0.05, **p < 0.01, ***p < 0.001. (PDF) [file ppat.1007493.s017.pdf]
