# Supplementary material for: Modeling the measles paradox reveals the importance of cellular immunity in regulating viral clearance
Source: PLoS Pathog. 2018 Dec 28;14(12):e1007493. doi: 10.1371/journal.ppat.1007493 (PMC6310241; doi:10.1371/journal.ppat.1007493)
Supplement: S2 Fig — Points indicate data for (A) total lymphocytes, (B) MV-specific T cells, and (C) viral load; solid lines indicate the corresponding model predictions determined by maximum likelihood optimization. The activated T cell predictions are depicted before scaling for comparison with the MV-specific T cell data. Each row corresponds to an individual macaque (with identification codes inset in panel C), and panels B and C are shown on the log scale. (PDF) [file ppat.1007493.s002.pdf]

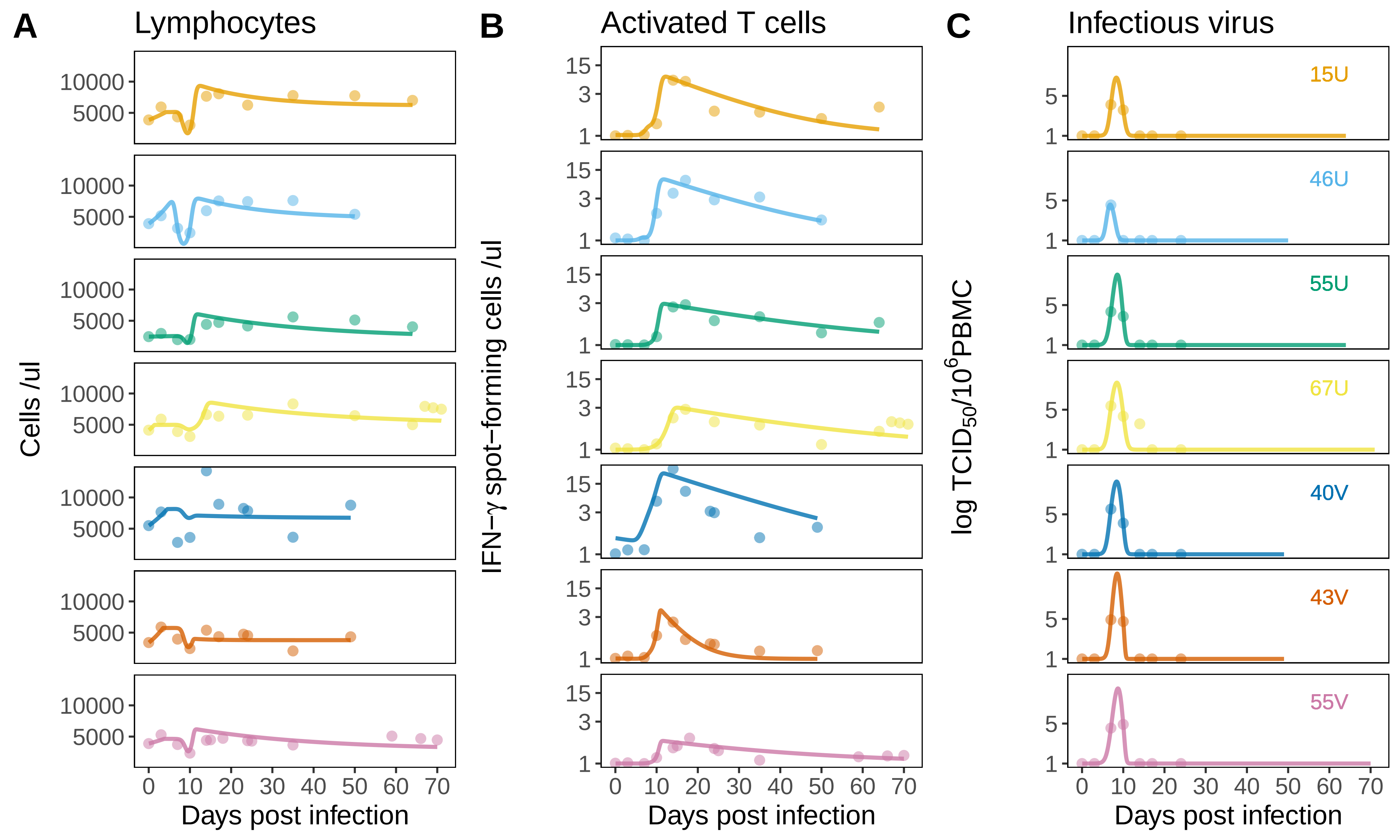

A
Lymphocytes
B
Activated T cells
C
Infectious virus
Cells /ul
IFN−γ spot−forming cells /ul
log TCID$_{50}$/10^{6}PBMC
15U
46U
55U
67U
40V
43V
55V
10000
5000
15
3
1
5
0 10 20 30 40 50 60 70
Days post infection
