# Supplementary material for: Modeling the measles paradox reveals the importance of cellular immunity in regulating viral clearance
Source: PLoS Pathog. 2018 Dec 28;14(12):e1007493. doi: 10.1371/journal.ppat.1007493 (PMC6310241; doi:10.1371/journal.ppat.1007493)
Supplement: S6 Fig — Fitted parameter estimates were obtained from 500 bootstrap samples. Colors and numerical values indicate the magnitude of correlation; correlations above a standard significance threshold (i.e. p > 0.05) are depicted in white. (PDF) [file ppat.1007493.s006.pdf]

55V

	A_0	β	k	p	ψ	q	q_s	r	s	t_d	V_0
A_0	1	−0.32	−0.17	0.3	−0.1	−0.57	0	−0.05	−0.07	0.03	−0.02
β		1	−0.48	−0.49	0.46	0.6	0.02	−0.03	0	−0.05	−0.33
k			1	0.53	−0.41	−0.07	0.02	−0.05	0.07	0.13	0.06
p				1	−0.38	−0.34	0.04	0	0.05	0.08	−0.02
ψ					1	0.19	−0.26	−0.15	−0.08	−0.08	−0.12
q						1	0	0.03	0.48	−0.01	−0.25
q_s							1	0.05	0.01	−0.22	0.03
r								1	−0.07	−0.06	0.1
s									1	0.04	−0.14
t_d										1	−0.06
V_0											1
