# Supplementary material for: Modeling the measles paradox reveals the importance of cellular immunity in regulating viral clearance
Source: PLoS Pathog. 2018 Dec 28;14(12):e1007493. doi: 10.1371/journal.ppat.1007493 (PMC6310241; doi:10.1371/journal.ppat.1007493)
Supplement: S1 Appendix — (PDF) [file ppat.1007493.s018.pdf]

Modeling the measles paradox reveals the importance of cellular immunity in regulating viral clearance

Sinead E. Morris*, Andrew J. Yates, Rik L. de Swart, Rory D. de Vries, Michael J. Mina, Ashley N. Nelson, Wen-Hsuan W. Lin, Roger D. Kouyos, Diane E. Griffin, Bryan T. Grenfell

*sinead.morris@columbia.edu

S1 Appendix

Experimental methods

Details of how the data (e.g. Fig 1) were generated are outlined briefly below; further information may be found in the original paper of Lin et al. (2012) [1].

Viral load

As measles virus (MV) infection causes a cell-associated viremia, viral load was determined as the number of productively infected cells per million peripheral blood mononuclear cells (PBMC). Using an infectious center assay, several replicates of multiple tenfold dilutions of PBMC were tested for their ability to infect susceptible cells. Cocultivation was screened between 4 and 7 days and a well was either scored as positive or negative for infection. The 50% point was then determined using the formula of Reed and Muench [2], and the result was normalized to the number of infected cells per million PBMC based on the number of cells used in the first dilutions.

Lymphocytes

We use the total number of lymphocytes as a proxy for the main virus target cells. Lymphocyte counts were always collected in the morning, and the total number of lymphocytes per microliter of blood was measured using an automated cell counter. NK and NKT cells are not (or at best poorly) susceptible to MV infection and are not expected to contribute substantially to this population. In addition, monocytes and granulocytes were distinguished as separate populations. Thus the majority of the total lymphocyte counts in our data represent cells susceptible to infection.

MV-specific T cells

The IFN-γ spot forming cells (SFCs) were determined after MV-specific stimulation, and data shown are specific counts after the subtraction of counts obtained following mock stimulation. The data therefore represent antigen-specific cells and are used as a proxy for MV-specific activated T cells.

Avidity

Avidity was measured by enzyme immunoassay using plates coated with lysates from MeV-infected cells. An avidity index was calculated by the ability of MeV-specific plasma IgG to be eluted by increasing concentrations of NH4SCN (see for example [3]).

Alternative model formulations

T cell activation

The timing and magnitude of T cell activation is modeled in the main text using a continuous saturation function described previously for LCMV [4]. However, we also investigated alternative functional forms. First, we defined activation within a discrete timeframe such that

$$f(V) = \begin{cases} 1 & \text{if} \quad t_1 \leq t \leq t_2, \\ 0 & \text{otherwise,} \end{cases}$$

with all other model equations remaining unchanged. Note that this function has two parameters that must be estimated (t_1 and t_2), compared to one parameter for the continuous saturation function (s). Second, we investigated activation that was proportional to viral load, i.e. we set the function governing T cell proliferation in Eq 3 to $f(V) = V$, and all other terms containing $1 - f(V)$ to 1. This formulation has no additional parameters to estimate and results in the following system of equations:

$$\begin{aligned} \frac{dS}{dt} &= -\beta SV + q_s\hat{\delta}(t)S + rA \\ \frac{dI}{dt} &= \beta(S + A)V - \delta I - kIA \\ \frac{dA}{dt} &= qVA - \beta AV - (d + r)A \\ \frac{dV}{dt} &= pI - cV. \end{aligned}$$

Lymphocyte proliferation

Proliferation of general susceptible lymphocytes (S) is modeled in the main text using a discrete on/off function to describe early (but temporary) lymphocyte activation i.e. $q_s\hat{\delta}(t)S$, where

$$\hat{\delta}(t) = \begin{cases} 1 & \text{if} \quad t < t_d, \\ 0 & \text{if} \quad t \geq t_d. \end{cases}$$

Here, proliferation is controlled by two parameters: the rate of proliferation, q_s, and the duration of proliferation, t_d. However, we also investigated two alternative functional forms with fewer parameters. First, we defined proliferation to be constant and solely dependent on the parameter q_s by setting $\hat{\delta}(t) = 1 \; \forall \; t$. Second, we removed the proliferation term entirely by defining $\hat{\delta}(t) = 0 \; \forall \; t$.

Cell to cell transmission

In addition to our original model including a free variable for viral load, we also explored the capacity of cell-to-cell transmission models to explain the available data. First, we formulated the standard mass action model as follows:

$$\frac{dS}{dt} = -\beta SI + q_s\hat{\delta}(t)S + r(1 - f(I))A \tag{S1}$$

$$\frac{dI}{dt} = \beta(S + A)I - \delta I - kIA \tag{S2}$$

$$\frac{dA}{dt} = qf(I)A - \beta AI - (1 - f(I))(d + r)A, \tag{S3}$$

(S4)

where

$$f(I) = \frac{I}{s + I} \tag{S5}$$

for some saturation constant, s, and $\hat{\delta}(t)$ is as defined in the main text.

Second, we explored an alternative cell-to-cell transmission model that includes a correction factor, $f_c(I)$, to account for spread within a spatially confined region [5]. Such a model would be more applicable to dynamics occurring in a stationary environment, such as the lymphoid tissues, rather than that of the blood. The equations for this model are identical to Eqs S1–S5, except the transmission parameter β is multiplied by $f_c(I)$, where

$$f_c(I) = \begin{cases} 1 & \text{if} \quad I \leq m, \\ a(I-m)^3 + b(I-m)^2 + 1 & \text{if} \quad m < I < m + z, \\ \frac{\sqrt{m^2 + 8mI - 8m} - m}{2I} & \text{if} \quad I \geq m + z; \end{cases}$$

m is the number of neighbors of each cell (assuming spread within a two-dimensional lattice); and z, a, b are constants defined to maintain smoothness of f_c $\forall$ I (see Kumberger et al. (2018) for further information [5]).

For both these cell-to-cell transmission models, the viral load data were used to fit the infected cell compartment (I), the total lymphocyte data to fit the sum of the model lymphocyte compartments ($S + I + A$), and the MV-specific T cell data to fit the activated T cell compartment (A). We fit the model over a range of possible values for m (4, 6, and 8), and constant values for z, a, b were assigned following Kumberger et al. (2018).

Fitting procedure

For each individual and model, let n_X be the number of measurements of variable X, where X is either total lymphocyte counts (L), viral load (V), or MV-specific activated T cells (A). The residual sum of squares between the log-transformed data (D) and model predictions (M) is then given by

$$RSS_X = \sum_{i=1}^{n_X} \left[\ln\left(X_i^M\right) - \ln\left(X_i^D\right) \right]^2.$$

As outlined in Hogan et al. (2015), assuming normally-distributed residuals gives the following log-likelihood expression for X

$$\ln \hat{L}_X = -n_X \ln\left(\sqrt{2\pi}\sigma_X\right) - \frac{RSS_X}{2\sigma_X^2}. \tag{S6}$$

The maximum likelihood estimate of σ_X^2 is then obtained by setting

$$\partial_{\sigma_X} \hat{L}_X = -\frac{n_X}{\sigma_X} + \frac{RSS_X}{\sigma_X^3} = 0,$$

so that

$$\hat{\sigma}_X^2 = \frac{RSS_x}{n_X}.$$

Substituting this into Eq S6 then gives

$$\ln \hat{L}_X = -\frac{n_X}{2} \ln\left(\frac{2\pi}{n_X} RSS_X\right) - \frac{n_X}{2},$$

and combining all three measurement variables gives the full log-likelihood

$$\ln \hat{L} = -\frac{n_V}{2} \ln\left(\frac{2\pi}{n_V} RSS_V\right) - \frac{n_A}{2} \ln\left(\frac{2\pi}{n_A} RSS_A\right) - \frac{n_L}{2} \ln\left(\frac{2\pi}{n_L} RSS_L\right) - \frac{(n_V + n_A + n_L)}{2}.$$

Uncertainty and sensitivity analyses

Uncertainty analysis was carried out using Latin Hypercube sampling (LHS), which assesses the variability in model outcome caused by changes in input parameters. The general idea is to randomly draw values for all input parameters from predefined probability distributions without replacement. Each sample of new draws (spanning the entire parameter set) is then used to re-simulate the model and calculate the new output, usually defined as some representative summary measure (e.g. peak viral load, total viral load, cumulative number of infection events etc). For N new parameter sets, one then has a distribution of N new outputs that can be used to assess model variability arising from parameter uncertainty. Previous work suggests N should satisfy $N > 4/3K$, where K is the number of input parameters [6].

In this study we used total viral load to describe model output, given its relevance to the experimental simulations comparing drivers of viral clearance. To explore model predictions regarding viral resurgence, we conducted additional analysis using the time to secondary viral rebound (measured in days post infection) as a supplemental measure. To avoid setting prior assumptions on parameter values, we defined uniform distributions for all input parameters; we also chose $N = 100$ (which satisfies $100 > 4/3 \times 10 = 13.33$). Note that the scaling factor, ψ, was not included in the analysis as it does not appear in the model equations and does not directly impact the resulting simulations. Bounds on fitted parameter distributions were defined as the maximum and minimum values of the corresponding fitted estimates across all individuals; bounds on fixed parameters were defined as follows: $c \in (1, 6), d \in (1/80, 1/20), \delta \in (1/4, 1)$. Widening these bounds did not impact our overall conclusions. All simulations were performed using the target cell and T cell model described by Eqs 1–6.

To assess model sensitivity to individual parameters, the above uncertainty analysis was extended using partial rank correlation (PRC) methods. In brief, the PRC coefficient of each input parameter was determined by correlating the LHS draws of that parameter with the corresponding model output (whilst controlling for all other parameters). Algorithms for calculating PRC coefficients are described in detail elsewhere (e.g. [6]). The sign of the PRC coefficient indicates the direction of the impact on model outcome (e.g. a positive correlation coefficient indicates an overall increase in the summary measure), whereas the magnitude indicates the strength of the impact. This technique therefore highlights the parameters with the greatest influence on model outcome. In summary, although the described LHS and PRC methods sample a range of possible model outcomes, rather than providing absolute bounds on the fitted model trajectories, they are powerful tools for assessing and visualizing the impact of parameter uncertainty on model predictions [6].

References

1. Lin WHW, Kouyos RD, Adams RJ, Grenfell BT, Griffin DE. Prolonged persistence of measles virus RNA is characteristic of primary infection dynamics.

Proceedings of the National Academy of Sciences. 2012;109(37):14989–14994. doi:10.1073/pnas.1211138109.

2. Reed LJ, Muench H. A simple method of estimating fifty per cent endpoints. American Journal of Epidemiology. 1938;27(3):493–497. doi:10.1093/oxfordjournals.aje.a118408.

3. Nair N, Gans H, Lew-Yasukawa L, Long-Wagar AC, Arvin A, Griffin DE. Age-dependent differences in IgG isotype and avidity induced by measles vaccine received during the first year of life. The Journal of Infectious Diseases. 2007;196(9):1339–1345. doi:10.1086/522519.

4. de Boer RJ, Oprea M, Antia R, Murali-Krishna K, Ahmed R, Perelson AS. Recruitment times, proliferation, and apoptosis rates during the CD8(+) T-cell response to lymphocytic choriomeningitis virus. Journal of Virology. 2001;75(22):10663–9. doi:10.1128/JVI.75.22.10663-10669.2001.

5. Kumberger P, Durso-Cain K, Uprichard SL, Dahari H, Graw F. Accounting for space – quantification of cell-to-cell transmission kinetics using virus dynamics models. Viruses. 2018;10(4):E200. doi:10.3390/v10040200.

6. Blower SM, Dowlatabadi H. Sensitivity and uncertainty analysis of complex models of disease transmission: an HIV model, as an example. International Statistical Review. 1994;62(2):229–243. doi:10.2307/1403510.

7. Hogan T, Gossel G, Yates AJ, Seddon B. Temporal fate mapping reveals age-linked heterogeneity in naive T lymphocytes in mice. Proceedings of the National Academy of Sciences. 2015;112(50):E6917–E6926. doi:10.1073/pnas.1517246112.
