# Supplementary material for: Modeling the measles paradox reveals the importance of cellular immunity in regulating viral clearance
Source: PLoS Pathog. 2018 Dec 28;14(12):e1007493. doi: 10.1371/journal.ppat.1007493 (PMC6310241; doi:10.1371/journal.ppat.1007493)
Supplement: S1 Table — Each row represents a different individual and columns represent different model structures (in order of increasing complexity from left to right). For each individual, numerical values indicate the difference in AICc between each model and the model with the lowest AICc (and hence best statistical support). Zero values (in bold) therefore indicate the best-supported model. (PDF) [file ppat.1007493.s019.pdf]

Table S1. Comparison of alternative T cell activation functions using AIC_c.

Individual	Proportional to viral load	Continuous saturation	Discrete timeframe
**15U**	42.6	**0.0**	17.5
**46U**	6.4	**0.0**	7.5
**55U**	49.1	**0.0**	41.8
**67U**	9.8	**0.0**	14.4
**40V**	39.0	**0.0**	35.9
**43V**	53.8	**0.0**	38.0
**55V**	19.4	**0.0**	9.7

Each row represents a different individual and columns represent different model structures (in order of increasing complexity from left to right). For each individual, numerical values indicate the difference in AIC_c between each model and the model with the lowest AIC_c (and hence best statistical support). Zero values (in bold) therefore indicate the best-supported model.
