# Supplementary material for: Modeling the measles paradox reveals the importance of cellular immunity in regulating viral clearance
Source: PLoS Pathog. 2018 Dec 28;14(12):e1007493. doi: 10.1371/journal.ppat.1007493 (PMC6310241; doi:10.1371/journal.ppat.1007493)
Supplement: S2 Table — Each row represents a different individual and columns represent different model structures (in order of increasing complexity from left to right). For each individual, numerical values indicate the difference in AICc between each model and the model with the lowest AICc (and hence best statistical support). Zero values (in bold) therefore indicate the best-supported model. (PDF) [file ppat.1007493.s020.pdf]

Table S2. Comparison of alternative general lymphocyte proliferation functions using AIC$_c$.

Individual	No proliferation	Constant proliferation	Temporary proliferation
**15U**	**0.0**	43.5	41.3
**46U**	**0.0**	5.3	18.0
**55U**	**0.0**	12.8	35.2
**67U**	6.1	**0.0**	3.4
**40V**	**0.0**	23.9	43.4
**43V**	**0.0**	17.7	5.2
**55V**	**0.0**	29.8	50.6

Each row represents a different individual and columns represent different model structures (in order of increasing complexity from left to right). For each individual, numerical values indicate the difference in AIC$_c$ between each model and the model with the lowest AIC$_c$ (and hence best statistical support). Zero values (in bold) therefore indicate the best-supported model.
