# Supplementary material for: Modeling the measles paradox reveals the importance of cellular immunity in regulating viral clearance
Source: PLoS Pathog. 2018 Dec 28;14(12):e1007493. doi: 10.1371/journal.ppat.1007493 (PMC6310241; doi:10.1371/journal.ppat.1007493)
Supplement: S3 Table — Each row represents a different individual and columns represent different model structures (in order of increasing complexity from left to right). For each individual, numerical values indicate the difference in AICc between each model and the model with the lowest AICc (and hence best statistical support). Zero values (in bold) therefore indicate the best-supported model. (PDF) [file ppat.1007493.s021.pdf]

Table S3. Model comparisons using AIC$_c$ when general lymphocyte proliferation is omitted (i.e. $\hat{\delta}(t) = 0 \ \forall \ t$).

Individual	(i) Target cells	(ii) Target cells and T cells	(iii) Target cells, T cells, and antibodies
**15U**	123.6	**0.0**	16.0
**46U**	56.3	**0.0**	3.1
**55U**	105.1	**0.0**	5.2
**67U**	5.3	**0.0**	4.1
**40V**	120.5	**0.0**	15.2
**43V**	110.9	6.80	**0.0**
**55V**	110.9	**0.0**	2.6

Each row represents a different individual and columns represent different model structures (in order of increasing complexity from left to right). For each individual, numerical values indicate the difference in AIC$_c$ between each model and the model with the lowest AIC$_c$ (and hence best statistical support). Zero values (in bold) therefore indicate the best-supported model.
