# Supplementary material for: Modeling the measles paradox reveals the importance of cellular immunity in regulating viral clearance
Source: PLoS Pathog. 2018 Dec 28;14(12):e1007493. doi: 10.1371/journal.ppat.1007493 (PMC6310241; doi:10.1371/journal.ppat.1007493)
Supplement: S4 Table — Each row represents a different individual and columns represent different model structures (in order of increasing complexity from left to right). For each individual, numerical values indicate the difference in AICc between each model and the model with the lowest AICc (and hence best statistical support). Zero values (in bold) therefore indicate the best-supported model. For the majority of individuals, the best supported model includes infectious (free) virus. (PDF) [file ppat.1007493.s022.pdf]

Table S4. Comparison of alternative transmission kinetics using $\mathbf{AIC}_c$.

Individual	Cell-to-cell	Infectious virus
**15U**	15.5	**0.0**
**46U**	**0.0**	93.0
**55U**	21.6	**0.0**
**67U**	**0.0**	14.8
**40V**	4.0	**0.0**
**43V**	44.3	**0.0**
**55V**	10.7	**0.0**

Each row represents a different individual and columns represent different model structures (in order of increasing complexity from left to right). For each individual, numerical values indicate the difference in AIC_c between each model and the model with the lowest AIC_c (and hence best statistical support). Zero values (in bold) therefore indicate the best-supported model. For the majority of individuals, the best supported model includes infectious (free) virus.
